# Universal behavior of hydrogels confined to narrow capillaries

**DOI:** 10.1038/srep17017

**Published:** 2015-11-24

**Authors:** Yang Li, Ozan S. Sarıyer, Arun Ramachandran, Sergey Panyukov, Michael Rubinstein, Eugenia Kumacheva

**Affiliations:** 1 Department of Chemical Engineering & Applied Chemistry, University of Toronto, Toronto; 2 Department of Chemistry, University of North Carolina, Chapel Hill, North Carolina 27599-3290; 3 P. N. Lebedev Physics Institute, Russian Academy of Sciences, Moscow 117924; 4 Department of Chemistry, University of Toronto, Toronto; 5 Institute of Biomaterials & Biomedical Engineering, University of Toronto, Toronto

## Abstract

Flow of soft matter objects through one-dimensional environments is important in industrial, biological and biomedical systems. Establishing the underlying principles of the behavior of soft matter in confinement can shed light on its performance in many man-made and biological systems. Here, we report an experimental and theoretical study of translocation of micrometer-size hydrogels (microgels) through microfluidic channels with a diameter smaller than an unperturbed microgel size. For microgels with different dimensions and mechanical properties, under a range of applied pressures, we established the universal principles of microgel entrance and passage through microchannels with different geometries, as well as the reduction in microgel volume in confinement. We also show a non-monotonic change in the flow rate of liquid through the constrained microgel, governed by its progressive confinement. The experimental results were in agreement with the theory developed for non-linear biaxial deformation of unentangled polymer gels. Our work has implications for a broad range of phenomena, including occlusion of blood vessels by thrombi and needle-assisted hydrogel injection in tissue engineering.

Properties of geometrically constrained synthetic and biological soft matter govern a large number of physical phenomena, including flow of complex liquids through narrow pores, confinement-induced phase transitions and shape transformations, wetting, adhesion and friction. In particular, flow of discrete soft matter objects through narrow quasi-one-dimensional capillaries or tubes is of great importance for industrial applications, e.g., in enhanced oil recovery^1^,^2^ and in biological and biomedical systems^3^,^4^,^5^,^6^,^7^,^8^,^9^,^10^,^11^,^12^,^13^,^14^,^15^,^16^,^17^,^18^,^19^. For example, blood clots can cause vascular occlusions and result in myocardial infarctions^8^, stroke^9^ and pulmonary embolism^10^,^11^. On the other hand, embolization of blood capillaries with soft polymer particles is utilized to reduce blood supply to tumor sites or treat endovascular aneurysm^13^,^14^,^15^,^16^. Furthermore, small nanometer-size conformable particles of biological matter can be cleared from the body by renal (glomerular) filtration through pores defined by narrow endothelial gaps^17^. Other exemplary systems include needle- or catheter-assisted injection of hydrogels for drug delivery^18^ and tissue engineering^19^.

While the systems listed above may belong to different classes of soft matter with a broad range of chemical and biophysical properties, upon their flow under quasi-one-dimensional environments, they have at least one feature in common: under a particular applied pressure they change their shape to enter and pass through a tube-like (capillary) environment with a diameter smaller than an unperturbed object size^20^. Establishing the underlying principles of the behavior of soft matter objects with different original, unperturbed dimensions and mechanical properties, which are confined to capillaries with different entrance angles and diameters under various applied pressures can shed light on many of the biological systems described above.

Hydrogel particles with well-defined dimensions, shapes and mechanical properties represent an excellent model system for studies of many soft matter objects in constrained environments. By examining the behavior of micrometer-size hydrogel particles (microgels) passing through microfluidic (MF) channels, important insights have been obtained on the relationship between the microgel mechanical properties^20^, shape^21^ and interactions with surfaces^22^,^23^,^24^ and microgel flow under confinement. The generalized relationship between the properties of soft objects, the geometry of constrained space and the behavior of soft objects in narrow capillaries are yet to be established.

In the current work, we report the results of experimental studies of the entrance, the passage and the change of volume of microgels entering narrow capillaries with diameters smaller than unconstrained microgel dimensions, as well as the flow of liquid through confined microgels. Importantly, in our work, the microgels were constrained in the capillaries under applied pressure difference from their swollen, unperturbed state, in contrast with previous studies, in which gels were formed directly in the narrow capillary by gelling a precursor solution^25^,^26^,^27^.

Theoretically, the “universal” behavior was demonstrated for gels formed by unentangled flexible polymer chains, cross-linked permanently on the time scale of the experiments, and swollen in good solvents. The theoretical results were validated in MF experiments conducted with agarose microgels introduced into narrow capillaries.

For the different degrees of confinement of microgels with varying mechanical properties in capillaries with different entrance angles we established experimentally and theoretically the underlying principles for (*i*) the flux of liquid through the confined microgel; (*ii*) the microgel’s location in the constriction under a particular applied pressure difference; (*iii*) the critical applied pressure difference beyond which the microgel passes the constriction and (*iv*) the loss of water by the confined microgel. Importantly, we show the relationship between these parameters and predict the behavior of the microgel in confinement using a theoretical model, which is in excellent agreement with experimental results.

This work has direct implications for the embolization of blood capillaries with hydrogel particles or for the needle-assisted injection of hydrogels for drug delivery and tissue engineering. For biological systems with a higher complexity, e.g., for thromboembolism or the flow of blood cells under confinement, our results may help in gaining understanding of the physical principles governing the system behavior. The predictive power of our results for such systems is yet to be tested.

## Results

### Microgel translocation through a capillary

**Figure 1:**
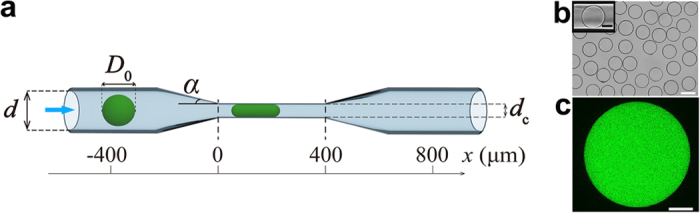
Schematic of the MF channel and unperturbed agarose microgels. (**a**) Schematic of the MF channel with a circular cross-section. The diameter, *d*, of the channel-at-large is 110 ± 5 μm, the diameter, *d*_c,_ of the narrow segment (constriction) is 43 ± 2 μm (α of 15°, 45°, or 60°) or 38 ± 2 μm (α = 30°). The length of the constriction is 400 μm. The abscissa *x* = 0 denotes the starting point of the constriction. A microgel with an unperturbed diameter of *D*_0_ moves along the direction indicated by the blue arrow, under the pressure difference applied along the MF device. (**b**) Representative optical microscopy image of 100 μm-diameter agarose microgels in an aqueous suspension. The scale bar is 100 μm. Inset shows a microgel with an unperturbed diameter of 80 μm in the channel-at-large (*d* = 107 μm). The scale bar is 40 μm. (**c**) Laser scanning confocal microscopy image of an agarose microgel (*D*_0_ = 115 μm) in the swollen state. The image was taken at the equatorial plane of the agarose microgel labeled with fluorescein isothiocyanate (FITC). The scale bar is 25 μm.

In a typical experiment, a microgel with a particular diameter and Young’s modulus was introduced in a microchannel comprising a channel-at-large with a diameter *d* of 110 ± 5 μm, a tapered section, and a constriction with a diameter *d*_c_ of 43 ± 2 μm or 38 ± 2 μm (Fig. 1a). The entrance angle, α, to the constriction was 15°, 45°, or 60°. We note that in a cardiovascular system, the tapering angle between wide and narrow blood vessels, that is, between a parent vessel and a daughter branch is typically, lower than 30°, but can reach 70° 28. The constriction expanded back to the channel-at-large with a diameter *d* of 110 ± 5 μm via the second tapered section. Both the channel-at-large and the constriction had circular cross-sections^29^. In Fig. 1a, the abscissa *x* = 0 denotes the entrance to the constriction. A microgel was forced into the microchannel by applying a pressure difference, Δ*P*, which was controlled by varying the relative heights of water reservoirs connecting upstream and downstream to the MF device (Supplementary Note 1). The motion of the microgel was recorded with a high-speed camera (Canon EX-F1) and analyzed by a code written in MATLAB.

Agarose microgels with an unperturbed diameter, *D*_0_, varying in the range from 40 to 120 μm and the Young’s modulus in the range from 2.6 to 20.2 kPa were prepared by the MF method described elsewhere^30^,^31^. Figure 1b shows a representative optical microscopy image of 100 μm-diameter agarose microgels. The particles had a round shape and 4.2% polydispersity (defined as the standard deviation in the dimensions of the microgels divided by the mean diameter). Inset shows an unperturbed 80 μm-diameter microgel in the channel-at-large.

We verified that polymer concentration is uniform throughout the body of the microgel by covalently labeling agarose molecules with a fluorescent dye fluorescein isothiocyanate and examining the distribution of fluorescence intensity in the microgel using confocal fluorescence microscopy images (Fig. 1c). Image analysis was conducted for the microgels in an unperturbed swollen state, where the effect of heterogeneity would be amplified^32^. The relative standard deviation in fluorescence intensity throughout the microgel body was 11.2 ± 0.9%, indicating that a swollen microgel has a uniform distribution in agarose concentration and structure on the micrometer scale.

When the microgel stopped at a particular pressure difference in the tapered region of the microchannel, the value of Δ*P* was incrementally increased in steps of 50 Pa and maintained for 60 s to stabilize the microgel at the new position along the *x*-axis. Following a repeated increase in Δ*P*, the microgel moved in a step-wise manner along the tapered zone, until it entered the constriction. At pressure difference Δ*P* exceeding Δ*P*_max_ the microgel passed through the constriction. A detailed description of these experiments is given in our previous work^33^.

**Figure 2:**
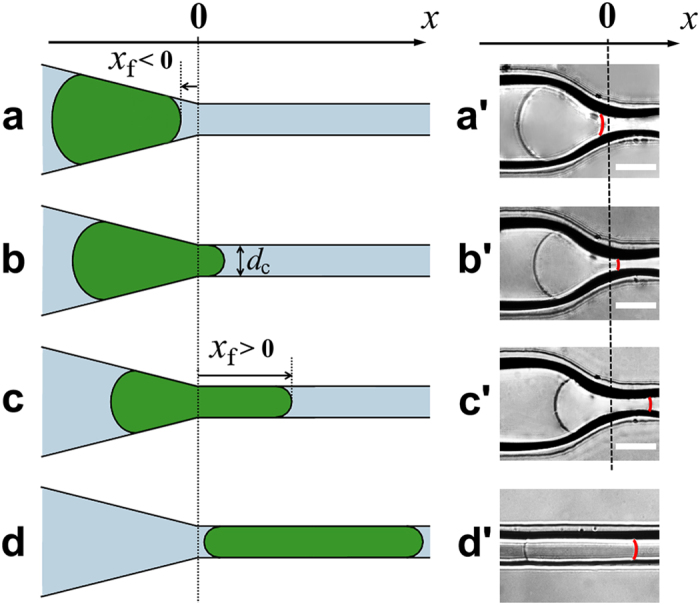
Microgel in different confined states. (**a–d**) Schematics of the microgel in different positions along the microchannel. (**a**) Microgel is localized completely in the tapered zone with the axial position of its front tip, *x*_f_ < 0. (**b**) A small portion of the microgel is located in the constriction of diameter *d*_c_, with a front tip position, *x*_f_ > 0. The pressure difference across the microgel is dominated by its tapered portion. (**c**) A large portion of the microgel enters the constriction, with the length *x*_f_ of the constricted microgel portion. The pressure difference across the microgel is dominated by its constricted portion. For *x*_f_ > 0 in (**b**) and (**c**), the smallest diameter of the microgel in contact with the microchannel walls is *d*_c_. (**d**) The microgel is localized completely in the constriction. (**a*****′*****–d*****′***) Representative optical microscopy images of the microgel in the confined states corresponding to (**a–d**). The red line shows the front tip of the microgel. α = 30°. The scale bar is 100 μm.

The shape of the spherical microgel changed under progressive confinement (Fig. 2). At small values of Δ*P*, the microgel body was localized completely inside the tapered region, with the axial position of its front tip *x*_f_ < 0 (Fig. 2a). In this case, the microgel acquired the shape of a truncated-cone with the left and the right caps facing the liquid. As the value of Δ*P* increased, the microgel front entered the constriction (*x*_f_ > 0) (Fig. 2b). The constricted part of the microgel acquired a capped cylindrical shape with a diameter *d*_c_. In this situation, as long as the length of the constricted part of microgel was relatively short, the pressure difference across the tapered microgel part exceeded the pressure difference along the constricted microgel portion. At higher Δ*P*, the value of *x*_f_ increased, and the pressure difference across the constricted part of the microgel exceeded the difference in pressure along the tapered microgel portion (Fig. 2c). The increase in *x*_f_ occurred, until Δ*P* exceeded Δ*P*_max_, at which the microgel completely entered the constriction and then passed it. In order to localize the microgel completely in the constriction (Fig. 2d), the value of Δ*P* was rapidly reduced to zero when the microgel was entirely in the narrow segment of the channel. The changes in the initial shape and volume of the microgel were reversible and completely recovered within 30 s after releasing it from the constriction^33^, and upon their repetitive insertion in the constriction, it exhibited identical behavior. Furthermore, for each Δ*P*, the change in the position and shape of the microgel was reversible and independent on the history of microgel insertion in the constriction. Figure 2a′–d′ show representative optical microscopy images of the microgel in the confined states (corresponding to Fig. 2a–d), with a distribution of microgel portions between the tapered region and the constriction.

### Flow of liquid through a confined microgel

As a microgel travels through the channel-at-large (*d* > *D*_0_), an insignificant water flow occurs through the polymer network, however when it is trapped in the tapered microchannel region, a pressure difference Δ*P* across the microgel creates significant water flux through it. For the microgel trapped at the entrance of the constriction, with the different portions of its body in the tapered zone and in the constriction, we performed fluorescence recovery after photobleaching (FRAP) experiments using confocal laser scanning microscopy (CLSM) to measure the rate of water flow, *Q*, as a function of Δ*P*. Microgels were suspended in 0.01 mg/mL aqueous solution of dextran molecules labeled with fluorescein isothiocyanate (average molecular weight 70,000 g/mol, and diameter^34^ 12.0 nm). After introducing a microgel into a microchannel, a pressure difference not exceeding Δ*P*_max_ was applied to the MF system to confine the microgel in the tapered zone of the microchannel. An intense laser pulse was used to photobleach a rectangular 250 μm × 150 μm region in the microchannel-at-large. To monitor the recovery of fluorescence, a series of 20 CLSM images was recorded at the attenuated beam intensity with 5 s intervals between image capturing. The flow rate of water was calculated from the change in the fluorescence intensity distribution between the images (Supplementary Notes 2 and 3).

**Figure 3:**
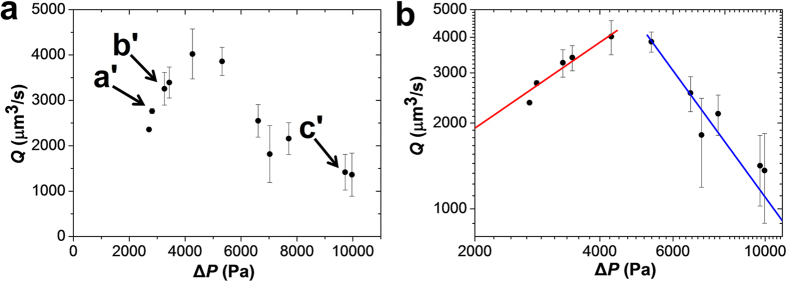
Effect of the pressure difference on the flow rate of water through the confined microgel. (**a**) Experimentally measured variation of the volumetric flow rate with pressure difference, plotted for the degree of confinement *D*_0_/*d*_c_ of 3.24 ± 0.03. Arrows indicate microgel positions a*′*–c*′* shown in Figs. 2a′ - 2c′. The portion of the microgel confined to the constriction increases with applied pressure difference. *D*_0_ = 104 μm, *d*_c_ = 38 μm, *E*_0_ = 2.57 kPa, α = 30°. (**b**) The same data presented on a log-log plot and compared with two scaling predictions *Q* ~ Δ*P* for weakly-deformed microgel (red line) and *Q* ~ Δ*P*^–2.0^ for constriction-dominated regime (blue line).

Figure 3a shows a non-monotonic variation of the flow rate *Q* with Δ*P*: an increase of *Q* at lower values of Δ*P*, followed by the reduction in water flow rate *Q* at higher values of Δ*P*. Arrows a′–c′ indicate microgel positions shown in Fig. 2a′–c′. We note that at Δ*P* < 2000 Pa, the contact between the microgel and the channel walls was not conformal and under these conditions, the flow rate of water was dominated by the leakage through the gaps between microchannel walls and the microgel (Supplementary Fig. S3). Significant leakage through the gaps was expected only at Δ*P* < *E*_0_, *E*_0_ the Young’s modulus of the undeformed gel (Supplementary Note 4).

The non-monotonic variation in the volumetric flow rate of water *Q*(Δ*P*) was explained as follows. In the absence of leakage, the flow rate of water through the microgel is equal to *Q* = Δ*P*/*R* (similar to Ohm’s law), where *R* is the hydraulic resistance of the porous microgel. Assuming laminar flow of water through the microgel pores, the hydraulic resistance of a pore of diameter ξ and length *L* is described by the Poiseuille’s law^35^ as *R*_ξ_ ~ η*L*/ξ^4^, where η is viscosity of water and the numerical coefficients are dropped. A microgel with length *L* and diameter *D* contains ~(*D*/ξ)^2^ such pores connected in parallel with a total hydraulic resistance




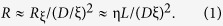




At small pressure difference Δ*P*, the spherical microgel is weakly deformed (*L*_0_ ≈ *D*_0_) and its resistance to flow *R*_0_ ~ η/(*D*_0_ξ_0_^2^) is almost independent of pressure. For this linear regime of weakly-deformed microgels, a linear dependence of the flow rate on the pressure difference is expected, that is, *Q* ≈ Δ*P/R*_0_, as shown with the red line in Fig. 3b, with the best fit value of resistance *R*_0_ ≈ 1 Pa·s/μm^3^ (Section 6 of Supplementary Note 5).

**Figure 4:**
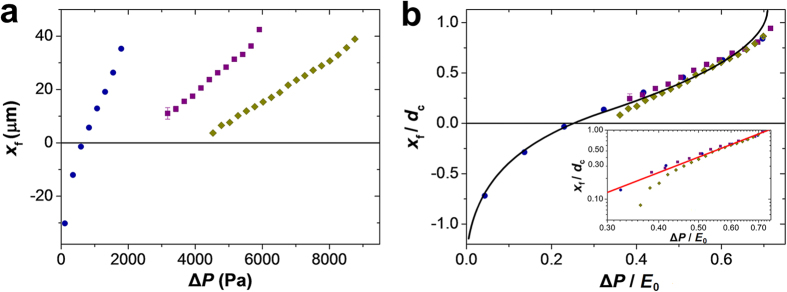
Dependence of the position of the microgel on the applied pressure difference. (**a**) Effect of pressure difference on the position of the microgel front tip *x*_f_ along the *x*-axis in the microchannel, plotted at the same degree of confinement *D*_0_/*d*_c_ = 2.24 ± 0.01 for the microgels with the Young’s modulus of 2.57 (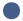), 8.26 (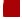) and 12.54 kPa (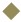) (corresponding to agarose concentration in the microgel of 2, 3, and 4 wt%, respectively). The results are obtained for microgels with corresponding diameters *D*_0_ of 94, 101, and 100 μm, which were passing through the constriction with a diameter *d*_*c*_ of 42, 45 and 45 μm, respectively, at α = 15°. (**b**) A master curve of the relative microgel position *x*_f_/*d*_c_, plotted as a function of Δ*P*/*E*_0_. The colors of symbols in (**b**) are the same as in (**a**). The black curve shows the result of numerical calculations with a fitting parameter κ = 0.365 (*see* Supplementary Fig. S7e). Inset shows the data for positive *x*_f_ on double-logarithmic scale. Red line with a slope 2.3 demonstrates agreement of data with scaling equations (2) and (6) for the constriction regime (*x*_f_ > 0).

At a higher pressure difference, the frictional force imposed by the flow of water pulls the microgel from the tapered zone into the constriction. In both regions, the microgel is biaxially compressed in the radial directions and elongated along the axial direction, however the shape of the microchannel makes this elongation non-uniform: the back end of the microgel in the tapered region is less elongated than its front end in the constriction. The position *x*_f_ of the front end of the microgel in the constriction is calculated by balancing the frictional force (imposed by the water flow on polymer chains) by osmotic force due to excluded volume repulsion and the elastic force due to the connectivity of the deformed polymer network, resulting in the following numerical result, which matches well with experiments.




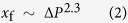




(*see* the red lines in Fig. 4b and Supplementary Fig. S7e). The osmotic pressure π originating from the excluded volume repulsion between the polymer chains of the strongly confined microgel is proportional to thermal energy *kT* per pore volume ξ^3^, where ξ is the correlation length in the constriction. The osmotic pressure π is determined by the applied pressure difference Δ*P* ≈ π ≈ *kT*/ξ^3^, and the pore size of the microgel decreases with increasing pressure as




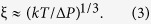




The strongly compressed portion of the microgel in the constriction dominates the flow resistance *R* that can be estimated from equation (1) as




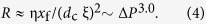




Here, the length of the flow resistance-dominating region is taken as the length of the constricted portion of the microgel *L* ≈ *x*_f_ (equation (2)), the width of the flow resistance-dominating region is the diameter of the constriction *D* ≈ *d*_c_, and the correlation length in constriction is approximated by equation (3). A strong increase of flow resistance with the applied pressure difference 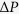 in the constriction-dominated regime (equation (4)) results in the decrease of flow rate




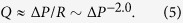




A sharp decrease of flow rate with increasing pressure difference (a blue line in Fig. 3b with the slope of −2.0) is in agreement with experimental observations. Thus the crossover from the tapering regime of weakly-deformed microgel with approximately constant flow resistance *R*_0_ and linear *Q –* Δ*P* relationship to the constriction regime of the strongly-confined microgel with flow resistance rapidly increasing with pressure difference (equation (4)) explains the non-monotonic pressure dependence of flow rate shown in Fig. 3.

### Microgel progression into confinement

With an increasing pressure difference Δ*P*, the microgel moves from the tapered zone into the constriction, as illustrated in Fig. 2. Figure 4a shows the change in the position of the front microgel tip, *x*_f_, plotted as a function of the applied pressure difference, Δ*P*, under the same degree of microgel confinement (defined as the ratio *D*_0_/*d*_c_). The negative values of *x*_f_ (Fig. 4a, left-most three blue symbols) correspond to the microgel located completely in the tapered region.

For the same degree of confinement, softer microgels with a lower Young’s modulus required a smaller pressure difference Δ*P* to move along the *x*-direction to enter the constriction. In Fig. 4b, the data from Fig. 4a are re-plotted in the normalized form: the position of the front microgel tip *x*_f_ was normalized by the constriction diameter *d*_c_, while the pressure difference Δ*P* was normalized by the Young’s modulus *E*_0_ of an unperturbed microgel of diameter *D*_0_. Under the same degree of confinement *D*_0_/*d*_c_, the three sets of data points obtained for the microgels with different moduli *E*_0_ overlapped to form a master curve for the variation in the relative microgel position *x*_f_/*d*_c_ *vs*. the normalized pressure difference Δ*P*/*E*_0_. The master curve illustrates that during microgel progression through the confinement, its position under a particular pressure difference was controlled by the rigidity and the size of the undeformed microgel.

The black curve in Fig. 4b shows the results of the numerical force balance calculation for the normalized position *x*_f_/*d*_c_ (a single fitting parameter κ = 0.365 was used to account for the neglected effects of out of plane bending of *y-z* slices of the microgel and the deformation of the microgel caps – *see* Supplementary Note 5). Figure 4b shows an excellent agreement between the numerical results and the experimental data.

Microgel progression from the tapered region to the constriction was rationalized as follows. When a large portion of the microgel was strongly constricted (Fig. 2c), the frictional forces *pulled* the constricted front portion of the microgel deeper into the constriction, while the wall force acting on the tapered back part balanced it. The force balance between the two portions of the microgel localized in the tapered and constriction regions led to equation (6) (a normalized form of equation (2))




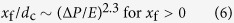




in the constricted regime (*see* Supplementary Fig. S7e). This scaling form, plotted as a red line in the inset of Fig. 4b, is in agreement with the experimental data for the significantly constricted microgel.

### Microgel passage through the constriction

At high pressure difference Δ*P* > Δ*P*_max_, the wall force acting on the tapered part of the microgel can no longer balance the friction force acting on the constricted part, and the microgel passes through the constriction (later in the text Δ*P*_max_ is referred to as a translocation pressure).

**Figure 5:**
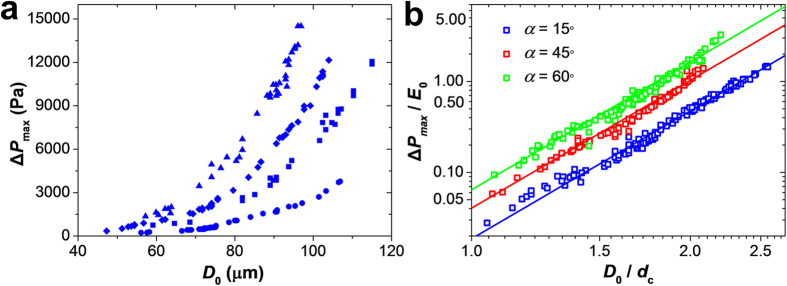
Dependence of Δ*P*_max_ on the degree of microgel confinement. (**a**) Variation in Δ*P*_max_ plotted as a function of microgel diameter *D*_0_. *d*_c_ = 43 ± 2 μm. Microgels with Young’s modulus of 2.57 kPa (circle symbols), 8.26 kPa (square symbols), 12.54 kPa (diamond symbols) and 20.21 kPa (triangle symbols) (corresponding to agarose concentration in the microgel of 2, 3, 4 and 5 wt%, respectively) were tested in the constriction with the entrance angle α = 15°. (**b**) Log-log plot of the master curves describing the relationship between Δ*P*_max_/*E*_0_ and the degree of microgel confinement *D*_0_/*d*_c_ in microchannels with different entrance angles α. Solid lines are the theoretical results obtained from equation (7) with a slope 14/3. Microgels with Young’s moduli (denoted as in (**a**)) were tested in constrictions with the entrance angles α of 15° (blue color), 45° (red color), and 60° (green color).

This effect was examined for the varying degrees of confinement of the microgels with different mechanical properties in microchannels with different entrance angles. Figure 5a shows the variation in Δ*P*_max_ *vs*. *D*_0_ for the microgels with different Young’s moduli *E*_0_ in the initial swollen and unperturbed state. For a particular value of *E*_0_, with increasing dimensions of the microgel, a greater pressure difference was required to force it into the constriction, owing to the stronger shape and volume change experienced by larger microgels. For a particular microgel diameter, the translocation pressure Δ*P*_max_ was greater for microgels with a higher value of Young’s modulus, since it was harder to deform a microgel with a higher stiffness. Figure 5b shows the dependence of the ratio Δ*P*_max_/*E*_0_ on the degree of microgel confinement *D*_0_/*d*_c_. For each geometry of microchannel studied, that is, the entrance angle α, all the data points collapsed onto a master curve, which combined four experimental parameters *D*_0_, *d*_c_, *E*_0_, and Δ*P*_max_. This result implies that for any set of three parameters *D*_0_, *d*_c_, *E*_0_, or Δ*P*_max_, the other characteristics can be obtained from the master curve. Importantly, the master curve could be used to measure the Young’s modulus of gel particles with different rigidity by examining their translocation pressure Δ*P*_max_ as a function of the degree of confinement.

Theoretically, the translocation pressure Δ*P*_max_ scales with the degree of confinement as




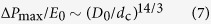




(the derivation of equation (7) and α-dependent coefficient of proportionality is given in Section 5b of Supplementary Note 5). The solid lines in Fig. 5b show the scaling form of equation (7) and match the experimental results.

Larger values of Δ*P*_max_/*E*_0_ were required to bring the microgel into the constriction with a larger tapering angle α (Fig. 5b). This result agreed with an earlier work performed for the limited range of entrance angles (3° and 6°), in which the difficulty in the transit of cells with an increasing entrance angle was established experimentally and theoretically^36^,^37^. With an increasing entrance angle α, the *x*-component of the wall force exerted on the tapered portion of the microgel increases proportional to 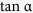, at small 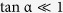, which requires a stronger friction force in the constricted part at the translocation point. Hence the pressure difference Δ*P*_max_ required to force the microgel into the constriction increases with the entrance angle α to the constriction.

### Change in microgel volume in the confined state

**Figure 6:**
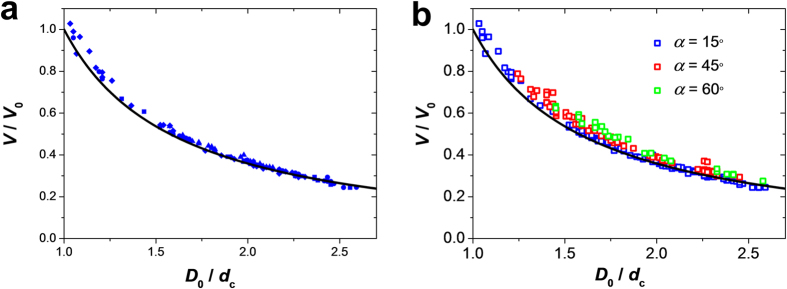
Effect of the degree of confinement on the change in microgel volume. (**a**) Ratio of the final-to-initial volume, *V/V*_0_ of the confined microgel, plotted as a function of the degree of microgel confinement, *D*_0_*/d*_c_. *V* is the volume of the microgel completely localized in the constriction and maintained there for 5 min. The Young’s moduli of the microgels are 2.57 (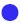), 8.26 (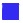), 12.54 kPa (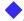), and 20.21 (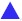) (corresponding to agarose concentration in the microgel of 2, 3, 4 and 5 wt%, respectively). The black line gives the theoretical result, equation (8), for *V/V*_0_. (**b**) Variation in volume ratio for microgels confined to the constrictions with different entrance angles.

Upon complete microgel entrance into the constriction, its equilibrium volume, *V*, at Δ*P* = 0, reduced in comparison with its original unperturbed volume, *V*_0_, implying that water was partly expelled from the microgel interior. We examined the change in microgel volume under confinement, *V*/*V*_0_, for the microgels with different Young’s moduli as a function of the degree of their confinement, *D*_0_/*d*_c_. Figure 6a shows that with an increasing degree of confinement, the ratio *V*/*V*_0_ decreased, due to a stronger compression of larger microgels and expulsion of a larger amount of water from their interior. Importantly, for the same values of *D*_0_/*d*_c_, the relative change in microgel volume was not affected by a ~10-fold change in Young’s modulus, implying that microgels with different deformabilities have the same Poisson’s ratio and the reduction in *V*/*V*_0_ exhibits the same dependence on the degree of confinement *D*_0_/*d*_c_. Since microgel rigidity was tuned by changing polymer content, we conclude that the relative reduction in microgel volume in confinement does not depend on polymer concentration and cross-linking density.

Importantly, a similar variation *V*/*V*_0_ *vs*. *D*_0_/*d*_c_ was observed for all microgels confined to a constriction with varying entrance angles (Fig. 6b). This similarity indicates that the values of *V* and *V*_0_ were measured in equilibrium states and the relative change of the microgel volume was independent on the history of microgel progression in the constriction.

We developed a quantitative model for the change in microgel volume in confinement, by considering a uniform equi-biaxial compression of a microgel, upon its complete entrance into the constriction. The increase in osmotic pressure in the microgel (due to the increase in polymer concentration) is partially relaxed by microgel elongation in the unconstrained direction. This elongation, in turn, creates an elastic stress in the axial direction. At an equilibrium state, the elastic stress built up in the polymer network is balanced by the osmotic pressure, which results in the relative volume decrease




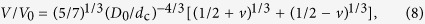




where 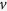 = [1/4–(8/4725) (*D*_0_/*d*_c_)^–10^]^1/2^ (Section 5a of Supplementary Note 5). These relations are represented by the black curves in Fig. 6a,b. The experimental and theoretical results were in excellent agreement without any fitting parameters. Comparison of the reduction in volume of the cylindrical and spherical microgels showed a ~10% difference in the variation of *V*/*V*_*0*_ with *D*_0_/*d*_c_.

## Discussion

In the Results section, we established experimentally and theoretically the underlying principles of the entrance, passage and confinement of polymer gels in narrow capillaries, as well as of the rate of flow through the confined gels. The position, the applied translocation pressure difference, and the change in volume of the confined gel, when normalized appropriately, depend only on the degree of confinement and the geometry of tapered region, and are independent of the polymer concentration in the gel in its undeformed state. The experimental and theoretical results were in excellent agreement.

Our model shows that these results are *universal* for unentangled gels formed by flexible polymer chains, swollen in a good solvent and cross-linked permanently on the time scale of experiments. As long as these criteria are met, the behavior of the gel does not depend on the chemical composition of the polymer or the solvent.

The non-monotonic effect of pressure on the flow rate of liquid through the geometrically constrained microgel is one of the most interesting and important findings of this work. The flux of water was determined by the location of microgel in the capillary and as such, was controlled by the applied pressure difference. At the moderate pressure drop, the flux of water was dominated by the almost constant hydraulic resistance of the microgel portion in the tapered confinement, and the flow rate of water increased with increasing pressure difference. As the microgel progressed further into the constriction region, the hydraulic resistance was rapidly increasing dominated by the constricted microgel part, and water flux reduced with increasing applied pressure difference.

The results of our work are relevant and important for the development of embolization therapies which utilize deliberate obstruction of blood vessels with gel beads and are used for treatment of hemorrhage or specific types of cancer^13^,^38^,^39^. In our work, the degree of confinement of microgels was from 1.1 to 2.4, that is, it was comparable with the range of 1.4 ± 0.38 to 3.1 ± 1.04 encountered in embolization therapies for occlusion of blood vessels^13^,^16^,^38^,^39^,^40^. On the other hand, there is no limitation in our model that precludes the use of larger degrees of confinement, both experimentally and theoretically, which underlines the universality of our model system.

Furthermore, our work offers a truly unique capability to mimic occlusion of blood capillaries in embolic strokes which account for ~25% of all strokes^41^. In embolic strokes, thrombi comprising fibrin gel travel from distant locations in the body to the brain, where they obstruct flow of blood in a narrow blood vessel. In the occluded vessel, the clot experiences deformation, loss of liquid and change in structure. These are the features reproduced in our model. According to the recent scientific evidence, changes in fibrin gel structure^42^, gel biomechanical properties^43^, and strain^44^ strongly influence the susceptibility of occlusive thrombi to thrombolysis. The microfluidic approach described in the present work offers the ability to study these effects. Finally, the non-monotonic change in flow rate of liquid through the occlusive microgel has far-reaching implications for the diffusion-driven *vs*. convection mediated mechanism of thrombolysis achieved by supplying a solution of a thrombolytic drug to the clot occluding a blood vessel^45^.

The theoretical approach developed in the present work can be extended to polymer gels that belong to other universality classes, including gels that are formed by (*i*) semi-flexible or rigid polymer chains, (*ii*) entangled chains, (*iii*) temporary cross-linkers, and (*iv*) gels swollen with theta-solvents or–in case of semi-flexible chains–marginal solvents. Based on the established dependence of elastic and osmotic energies on equilibrium polymer concentration, these gels will exhibit analogous trends under confinement, but with different scaling powers for the collapse of normalized data into master curves. For example, the change in volume upon confinement of unentangled gels formed by flexible chains in a theta solvent^46^ is expected to follow 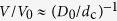, that is, with exponent *−1*, instead of *−4*/*3*, characteristic for gels swollen by a good solvent (*see* equation (8)).

We note that in confined gels, stresses are distributed over the entire gel volume. In contrast, for droplets or objects composed of a liquid core encapsulated by a membrane, e.g., vesicles and polymer capsules, deformation-induced stresses are manifested only at the interface^47^,^48^,^49^. An interface between a droplet and a continuous phase is described by interfacial tension, and for a particular degree of confinement, the translocation pressure difference will depend only on the geometry of the tapered region^47^. For vesicles and polymer capsules, interfacial stresses depend on multiple interfacial properties, e.g., bending, shear and area dilatation moduli, all of which would have to be accounted for, in order to collapse the normalized data into master curve^49^. Another important exception is the biological cell that can exhibit both active and passive (pressure-driven) mechanisms of passage through narrow capillaries, with its volume conserved during deformation^50^,^51^.

Importantly, the generalized relationship between the translocation pressure, the degree of microgel confinement and the entrance angle to the capillary enables the determination of the Young’s modulus of hydrogel particles, complementary to other techniques^52^,^53^, or the translocation pressure of hydrogel objects with known elastic properties. The latter feature result has implications in the biomedical field.

## Methods

### Preparation of agarose microgels

Agarose microgels with an unperturbed diameter, *D*_0_, varying in the range from 40 to 120 μm and the Young’s modulus, *E*_0_, in the range from 2.6 to 20.2 kPa were prepared by the microfluidic (MF) emulsification of an aqueous agarose solution at polymer concentration of 2, 3, 4, or 5 wt% and subsequent gelation of the precursor droplets at 4 °C for 20 min^30^,^31^. The Young’s moduli of the microgels were determined by the Atomic Force Microscopy method using a tipless cantilever, as described elsewhere^52^.

### Fabrication of microchannels with a circular cross-section

The rectangular cross-section of the microchannel, including the channel-at-large and the constriction, was transformed into a circular one by using a modification process described elsewhere^29^. First, a microchannel with a rectangular cross-section was fabricated in polydimethylsiloxane (PDMS) using a soft-lithography procedure^54^. The microchannel was filled with a solution of silicone oligomer in hexanes and a stream of N_2_ gas was injected into the microchannel. Upon heating, the oligomer was polymerized around the N_2_ stream. Following the evaporation of hexanes, the rectangular cross-section of both the channel-at-large and the constriction transformed into the circular cross-section with a diameter controlled by the pressure of N_2_ gas and the concentration of the solution of silicone oligomer in hexanes^29^.

### Experimental setup

Microgels were introduced into the MF device from a syringe connected to the upstream reservoir using a three-way valve. The pressure difference, Δ*P*, applied along the microchannel was controlled by varying the difference in heights (Δ*H*) of two water reservoirs connected upstream and downstream to the MF device (Supplementary Note 1). The motion of the microgel was recorded with a high-speed camera (Canon EX-F1) and analyzed by a code written in MATLAB.

### Flow of liquid through a confined microgel

We employed fluorescence recovery after photobleaching (FRAP) in combination with confocal laser scanning microscopy (CLSM) to measure the rate, *Q*, of water flow *through* a microgel trapped at the entrance to the constriction, as a function of applied pressure difference Δ*P*. Before introducing microgels into the MF channel, a 200 μm depth scanning experiments were performed at incremental steps of 5 μm along the *z*-direction of the microchannel to map a circular cross-section of the microchannel from the 3D reconstruction of the stacked CLSM images (Supplementary Fig. S1a,b). The FRAP experiments were performed along the *z*-direction at the middle position with the highest fluorescent intensity, in order to avoid the attenuation of intensity that occurs in CLSM experiments when *z*-coordinate is varied.

After introducing a microgel with an unperturbed diameter *D*_0_ = 104 μm into a microchannel (*d*_c_ = 38 μm, α = 30°), a pressure difference not exceeding Δ*P*_max_ was applied to the MF system to confine the microgel in the tapered zone of the microchannel. An intense (100% power of 5.4 mW), 25 s laser pulse photobleached a rectangular region in the microchannel-at-large using the NIS Element software. To monitor the recovery of fluorescence, the program set the instrument to the attenuated beam (10% power) and a series of 20 images was recorded with 5 s intervals between image capturing. Photoemission intensity data were collected from the images (selected images shown in Supplementary Fig. S2a), and the reference photoemission intensity was obtained from the image taken with the attenuated beam prior to the photobleaching event (Supplementary Notes 2 and 3).

## Additional Information

**How to cite this article**: Li, Y. *et al.* Universal behavior of hydrogels confined to narrow capillaries. *Sci. Rep.* **5**, 17017; doi: 10.1038/srep17017 (2015).

## Supplementary Material

Supplementary Information
