# Supplementary material for: Universal behavior of hydrogels confined to narrow capillaries
Source: Sci Rep. 2015 Nov 24;5:17017. doi: 10.1038/srep17017 (PMC4657056; doi:10.1038/srep17017)
Supplement: Supplementary Information [file srep17017-s1.pdf]

Supplementary Information to:

Universal behavior of hydrogels confined to narrow capillaries

Authors: Yang Li[1], Ozan S. Sarıyer[2], Arun Ramachandran[1], Sergey Panyukov[3], Michael Rubinstein[2*], Eugenia Kumacheva[1,4,5,*]

[1] Department of Chemical Engineering & Applied Chemistry, University of Toronto, Toronto. [2] Department of Chemistry, University of North Carolina, Chapel Hill, North Carolina 27599-3290. [3] P. N. Lebedev Physics Institute, Russian Academy of Sciences, Moscow 117924. [4] Department of Chemistry, University of Toronto, Toronto. [5] Institute of Biomaterials & Biomedical Engineering, University of Toronto, Toronto.

Supplementary Figures

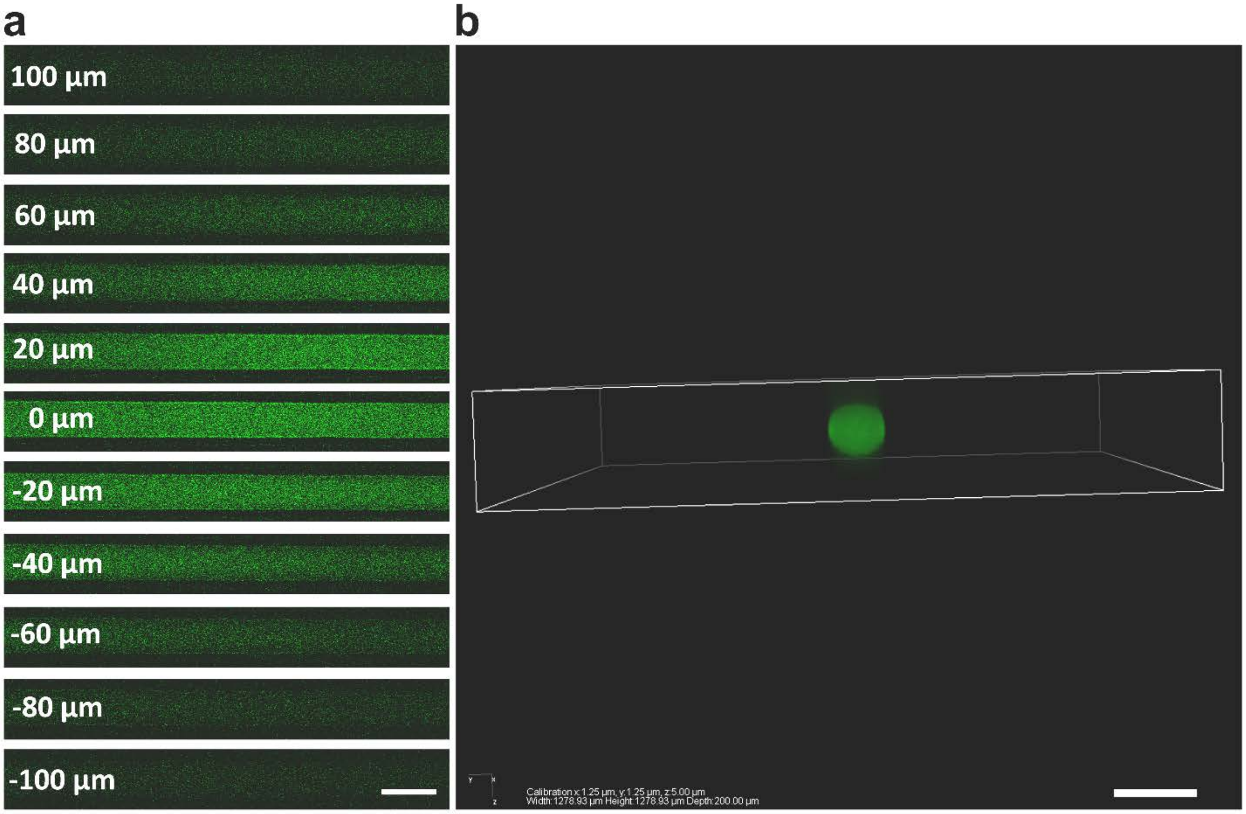

Supplementary Figure S1. Determination of the middle plane of a circular microchannel by using a *z*-stack of CLSM images. (a) Selected fluorescence microscopy images obtained by depth scanning of the microchannel filled with a 0.01 mg/mL of aqueous solution of fluorescein isothiocyanate-dextran by CLSM. A 200 μm-depth scanning was performed at an increment of 5 μm. The respective labels on the images show the position of the imaged plane with respect to the middle plane (labeled as 0 μm). FRAP experiments were conducted for the plane at z = 0 μm. **(b)** Circular cross-section of the channel-at-large obtained by 3D reconstruction of 41 images. Scale bar is 200 μm and applies to all images.

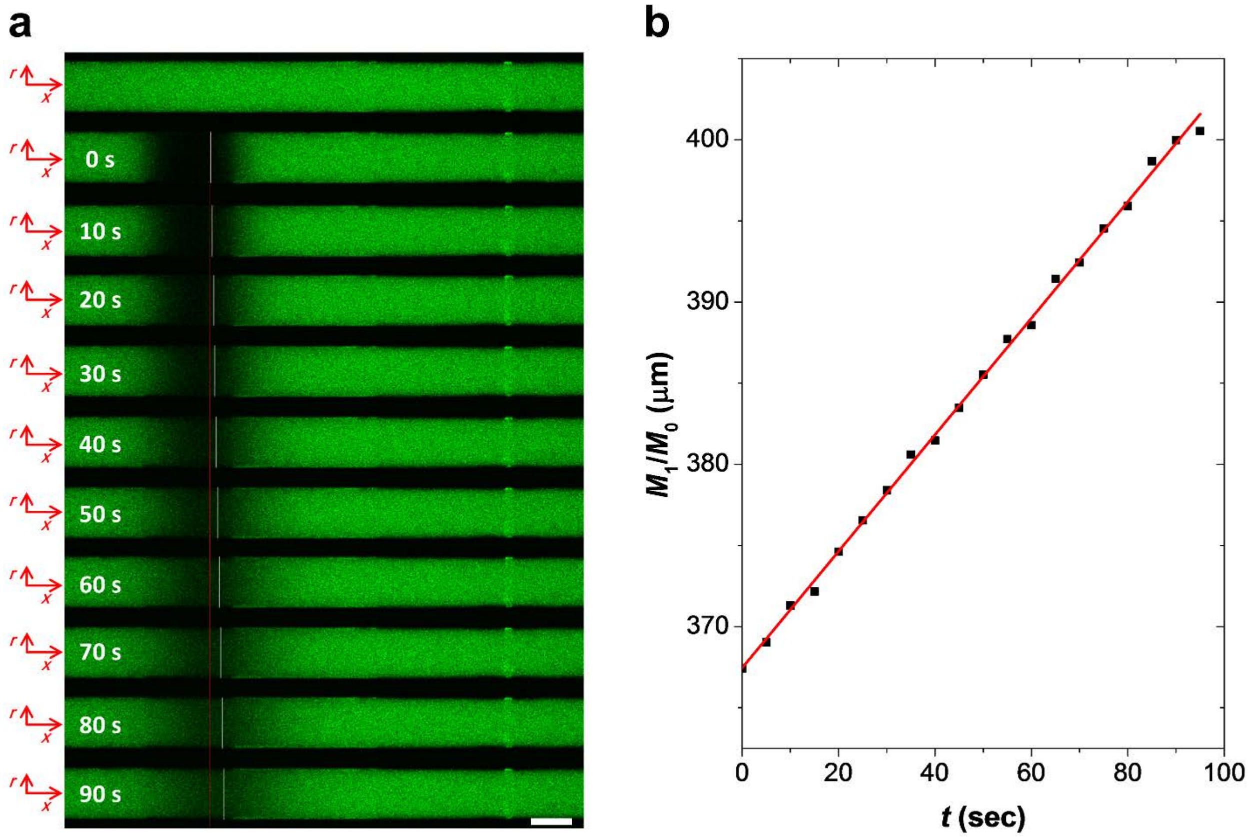

Supplementary Figure S2. Data analysis of FRAP experimental results. (a) A series of CLSM images obtained at 10% power of laser beam. The labels on the images show respective time after photobleaching event. The top image is captured before the photobleaching event at the same location. *x* is the flow direction and *r* is the radial coordinate of the circular cross-section. Scale bar is 100 μm. The calculated value of M_1/M_0 with time from **(b)** is labeled as a vertical white line in each image, with the red line indicating the value of M_1/M_0 in the image of t = 0 s. **(b)** The variation of M_1/M_0, the normalized first moment of the fluorophore intensity distribution in the microchannel (*see* equation (1)), plotted as a function of time. The solid line is a fit of M_1/M_0 *vs*. *t* to the linear function $M_1/M_0 = X_0 + Ut$; the average velocity of the liquid in this experiment is $U = 0.35$ μm/s.

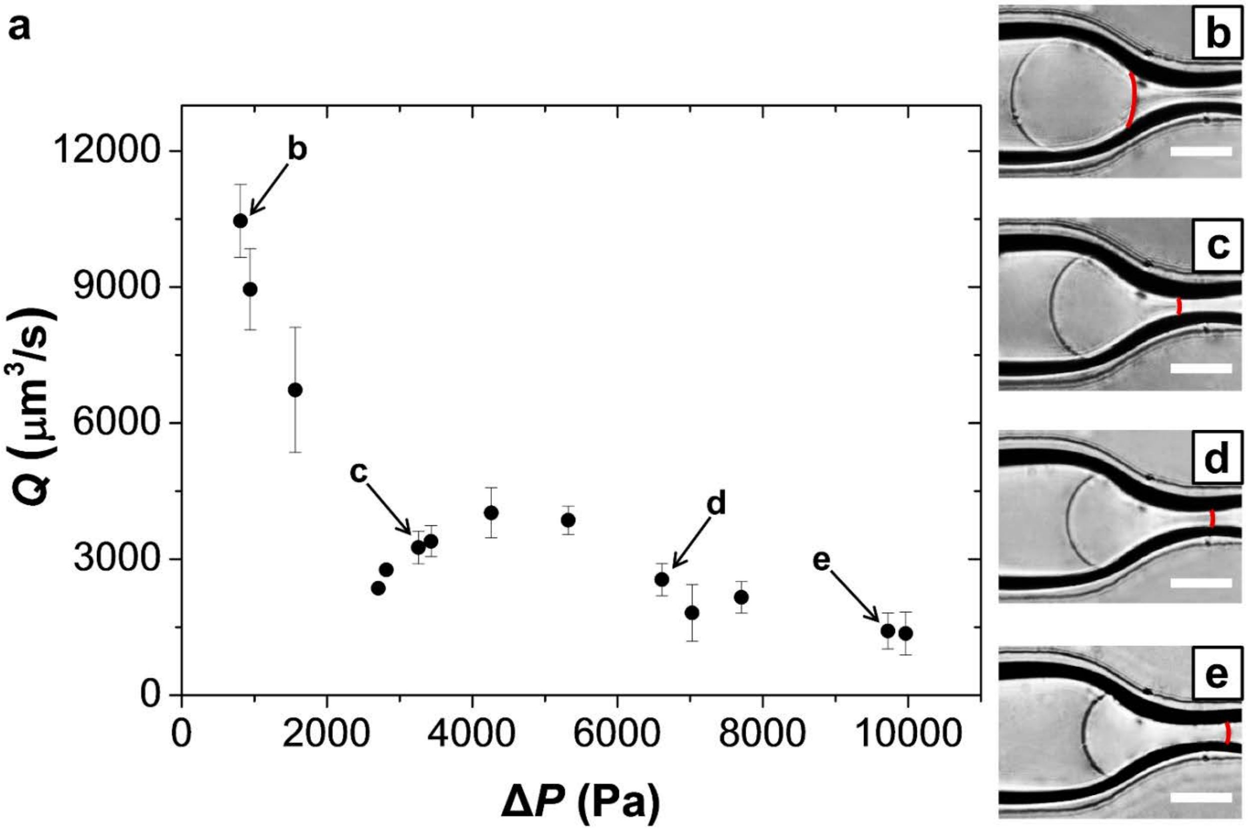

Supplementary Figure S3. Effect of pressure difference on the volumetric flow rate of water through the microgel. (a) Variation of the flow rate with the pressure difference, plotted for the confinement $D_0/d_c = 3.24 \pm 0.03$. **(b-e)** Optical microscopy images of the confined microgel under applied pressure difference of 808, 3258, 6610, and 9721 Pa, respectively. The red line in the images marks the front tip of the microgel. Scale bar is 100 μm. D_0 = 104 μm, d_c = 38 μm, E_0 = 2.57 kPa, α = 30°.

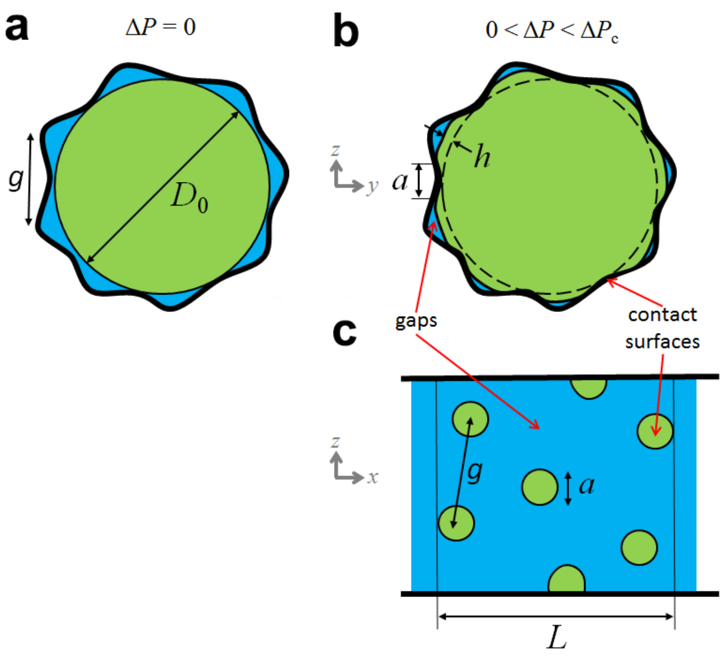

Supplementary Figure S4. Schematics of the leaking gaps. **(a-b)** Front view of the *yz*-cross-section of the microchannel and **(c)** contact spots on the surface of the microchannel. **(a)** At $\Delta P = 0$ the undeformed microgel barely touches the microchannel walls. Water can flow through the gaps of size *g*. **(b)** As ΔP increases, the microgel begins to conform to the microchannel surface, and **(c)** spots of contact areas grow on the microchannel surface.

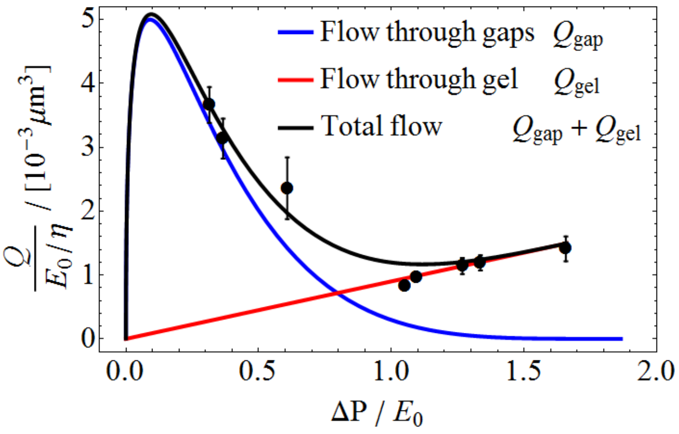

Supplementary Fig. S5. **Variation of *Q vs.* Δ*P* in the leakage-dominated and linear response regimes.** Pressure difference dependence of Q_{gap}, Q_{gel} and their sum Q fitted to experimental data (circles) in the leakage-dominated regime and the following linear response regime with the best fitting parameters g = 0.38 μm and $\Delta P_c/E_0$ = 1.9. Contributions to the total flow rate (black) from the flow through the leaking gaps (blue) and the microgel (red) are shown separately. Note that the leakage flow is insignificant in the higher pressure difference regime $\Delta P > E_0$ shown in Fig. 3 (main text).

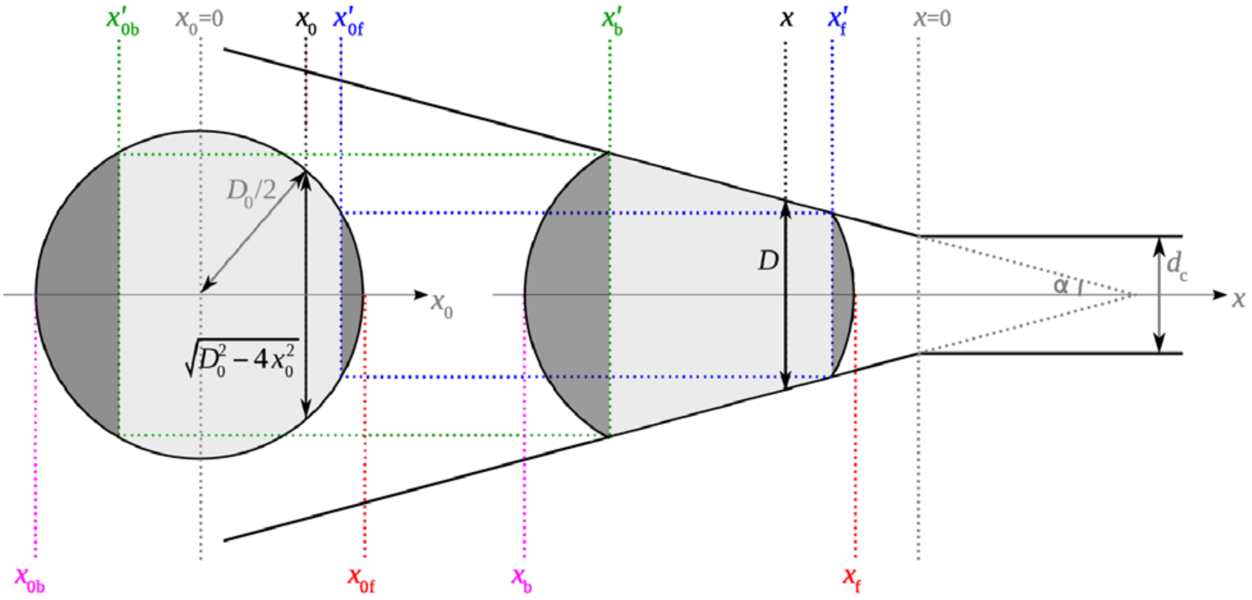

Supplementary Figure S6. Schematics of microgel confinement. Axial coordinates x_0 and x are used for the undeformed and deformed states of the microgel; $x_0 = 0$ corresponds to the center of the unperturbed microgel and $x = 0$ corresponds to the entrance of the constriction. We assume that a disk-shaped thin slice in undeformed state at position x_0 with respect to its center deforms homogeneously without bending into a disk-shaped thin slice at $x(x_0)$. Front and back microgel tip positions are x_f and x_b, respectively, while the front and back boundary positions of the deformed gel are x'_f and x'_b (axial boundaries of microgel portion in contact with the microchannel walls). We neglect the deformation of the back and front caps (dark shaded regions) assuming $\lambda_\perp = \lambda_\parallel = 1$ inside the caps ($x_b \leq x \leq x'_b$ and $x'_f \leq x \leq x_f$). With these boundary conditions, we numerically solve the differential force balance equations (55) and (56).

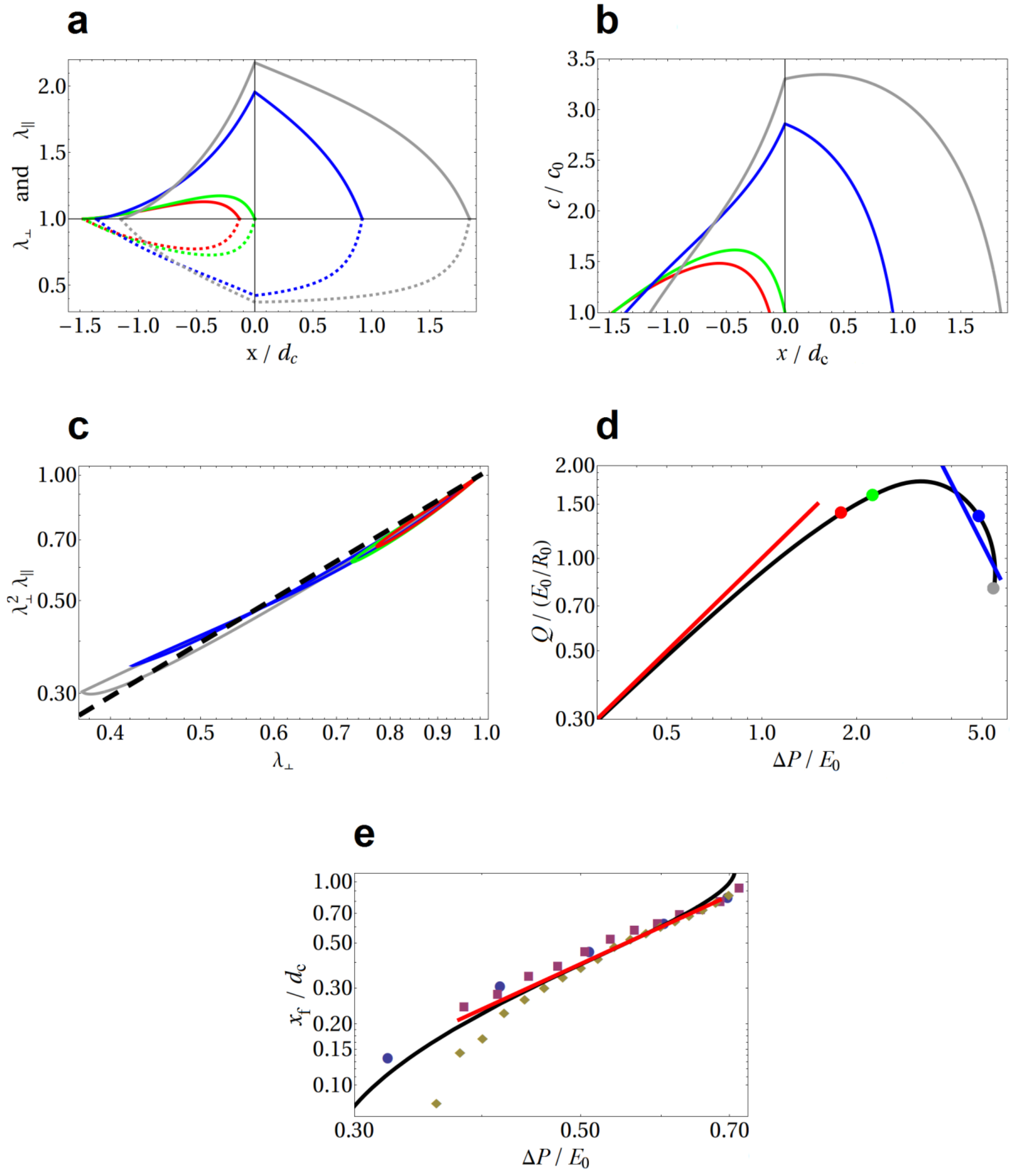

Supplementary Figure S7. Numerical results for the deformation ratio profiles, normalized concentration profiles, volume change factor, normalized flow rate, and normalized front tip position of the microgel in the constriction. Geometrical parameters are α = 30°, d_c = 38 µm, D_0 = 103

μm in **(a-d)** and $\alpha = 15°$, $d_c = 42$ μm, $D_0 = 94$ μm in **(e)**. Qualitatively similar results are obtained for different sets of geometrical parameters. **(a)** Deformation ratios (ratios of deformed to undeformed lengths) in axial $\lambda_{\parallel}$ (upper solid curves) and radial $\lambda_{\perp}$ (lower dotted curves) directions as functions of axial coordinate x along the deformed microgel normalized by the constriction diameter d_c. Different colors correspond to different microgel locations: microgel completely localized in the tapered region ($x'_f < 0$, red lines); microgel at the point of entry into the constriction ($x'_f = 0$, green lines); partially constricted before translocation ($x'_f > 0$, $\Delta P < \Delta P_{max}$, blue lines), and microgel at the translocation instability point ($\Delta P = \Delta P_{max}$, grey lines). The vertical lines at $x = 0$ mark the constriction entrance in **(a)** and **(b)**. **(b)** Concentration profile along the confined microgels. Colors correspond to the same microgel locations along tapered and constriction zones as in **(a)**. Note that for microgel partially in constriction (blue and grey lines) the maximum concentration is near the boundary between tapered zone and constriction. **(c)** Dependence of the relative change in volume $\lambda_{\perp}^2 \lambda_{\parallel}$, on the radial deformation ratio $\lambda_{\perp}$. Logarithmic axes. Colors correspond to the same microgel locations along tapered and constriction zones as in **(a)**. Black dashed line representing the asymptotic dependence $\lambda_{\perp}^2 \lambda_{\parallel} \sim \lambda_{\perp}^{4/3}$ (obtained from equation (63)) predicted for the case of no external axial stress is in reasonable agreement with the numerical solutions in the presence of external axial stress due to flow through the microgel (colored lines). **(d)** Dependence of the flux Q (normalized by E_0/R_0, *see* equation (73)) through the microgel on the pressure difference ΔP across it (normalized by E_0). Solid black curve shows numerical calculation described in Section 4, including flow through the caps. Red line shows asymptotic linear dependence $Q \sim \Delta P$ for low pressure differences. Blue line shows asymptotic scaling $Q \sim \Delta P^{-2.0}$ for high pressure differences. Colored circles correspond to the same microgel locations as in **(a)**-**(c)**. **(e)** Normalized front tip position of the microgel in the constriction x_f/d_c as a function of normalized pressure difference $\Delta P/E_0$. Black curve is the result of the numerical calculations for $\alpha = 15°$, $d_c = 42$ μm, $D_0 = 94$ μm. Markers show the experimental results (*see* Fig. 4b of the main text) for these geometrical parameters. The red line with a slope 2.3 (*see* equation (59)) is in good agreement with numerical and experimental results over a wide range of $\Delta P/E_0$ for the strongly constricted microgel. This scaling relation (equations (2) and (6) in the main text) is used in the theoretical analysis of experiments (equations (3-5) in the main text).

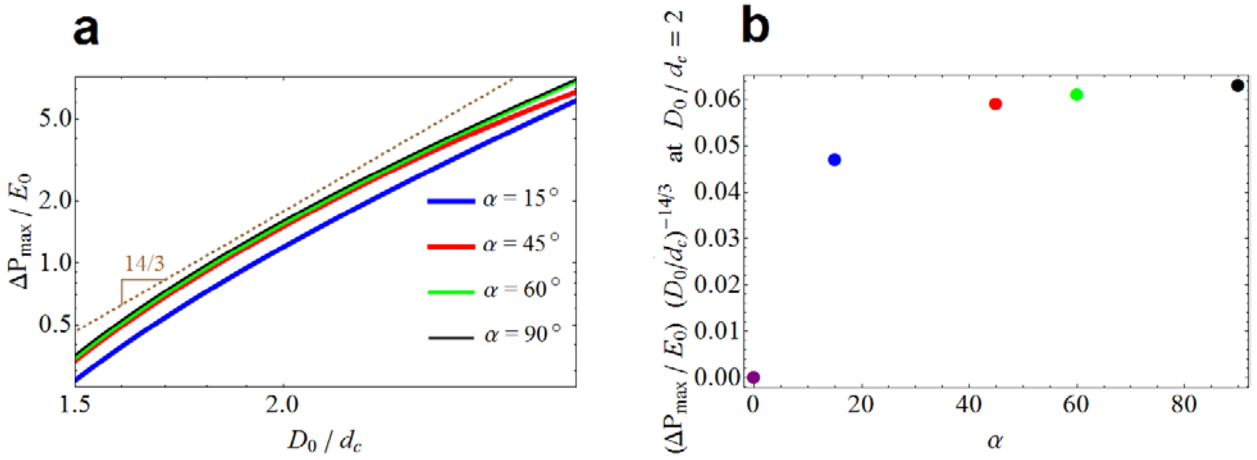

Supplementary Figure S8. Translocation pressure difference and angle-dependent amplitude *K*(α). **(a)** Simulation results for the rescaled translocation pressure difference, $\Delta P_{max}/E_0$, as a function of degree of confinement, D_0/d_c, for different tapering angles, α = 15° (blue), 45° (red), 60° (green), and 90° (black). The power-law dependence, $\Delta P_{max} \approx (D_0/d_c)^{14/3}$ (shown with the dotted line), is persistent for all angles. **(b)** Angle-dependent amplitude of this power-law, *K*(α), at degree of confinement $D_0/d_c = 2$ for the angles shown in **(a)**. Same colors are used to indicate different angles as in **(a)**. The amplitude *K*(α) saturates to a constant for large angles.

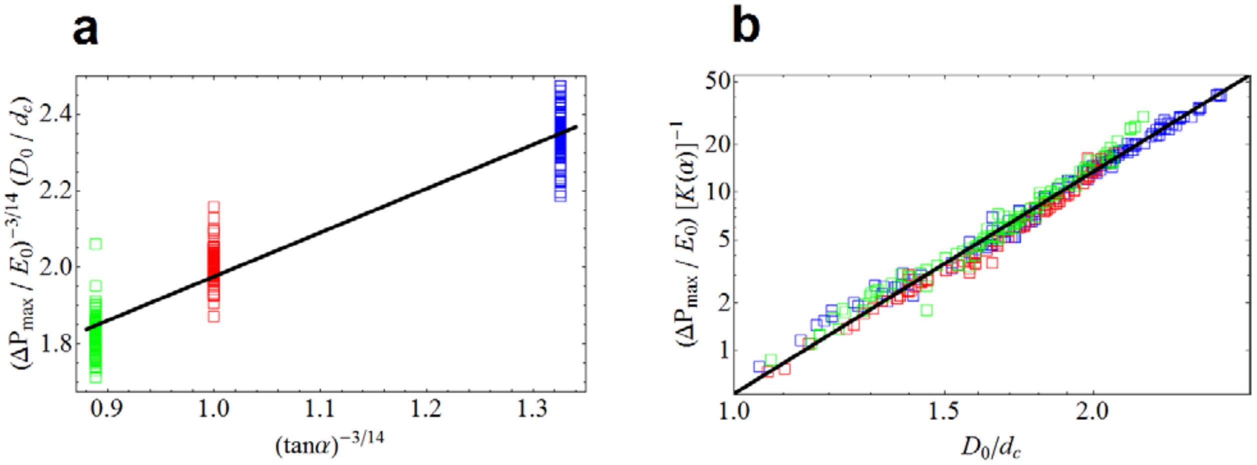

Supplementary Figure S9. Master curves for translocation pressure difference. (a) Experimental data shown in Fig. 5b plotted in the form $(\Delta P_{max}/E_0)^{-3/14}$ (D_0/d_c) as a function of $(\tan\alpha)^{-3/14}$. Data in this form is fit to the predicted function, $[K(\alpha)]^{-3/14} = k + (\tan\alpha)^{-3/14}$ (*see* equation (71)). The resulting best fitting parameter is $k = 0.72$ as shown with the black curve. **(b)** Experimental $\Delta P_{max}/E_0$ data rescaled with $K(\alpha)$ for $k = 0.72$, plotted *versus* D_0/d_c (in log-log axes). Data for all angles collapse on a single line with a slope 14/3 (shown in black). Both in **(a)** and **(b)**, different colors indicate different tapering angles: α = 15° (blue), 45° (red) and 60° (green), as in Fig. 5b and Supplementary Fig. S8.

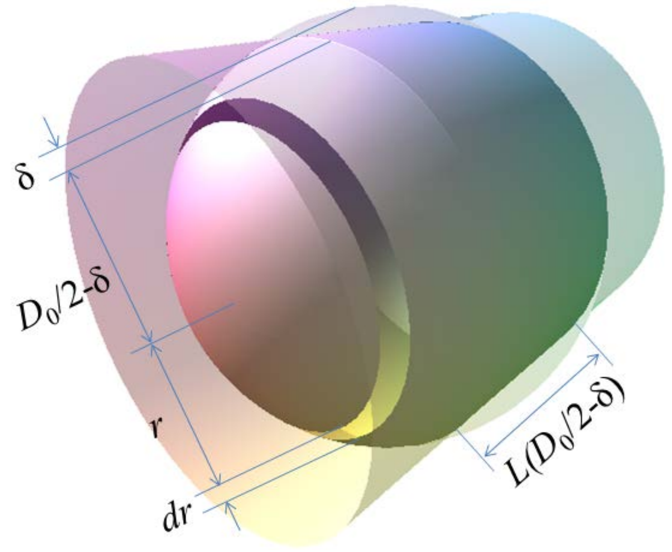

Supplementary Figure S10. Weak indentation by δ of a spherical microgel of diameter D_0 inside the tapered zone with the small angle α ≪ 1. The radius of the tapered microchannel in the contact zone is $D_0/2–\delta$. The axial length of the contact between microgel and microchannel is $L(D_0/2–\delta)=2\sqrt{\delta(D_0-\delta)}$ The resistance R is obtained by integration over cylindrical rings of radius r, thickness dr and length $L(r)$.

Supplementary Notes

Supplementary Note 1. Experimental details

1. Chemicals

Surfactant Span-80, mineral oil (viscosity of 30 cp), hexanes, and fluorescein isothiocyanate (FITC)-dextran (70 kD) were purchased from Sigma Aldrich (Canada) and used without further purification. FITC-dextran was stored at 4°C before its use in the experiments. Sylgard 184 Silicone elastomer kit containing poly(dimethylsiloxane) (PDMS) prepolymer and a crosslinker was obtained from Dow Corning Corp. (USA). Silicon wafers were acquired from Wafer World, Inc. (West Palm Beach, FL). SU-8 photoresist and SU-8 developer were supplied by MicroChem (USA). Ultralow gelling temperature agarose (SeaPrep) was obtained from Lonza (Switzerland).

2. Experimental setup

The experimental setup for microgel studies is described elsewhere[1]. Briefly, a MF device was interfaced with upstream and downstream water reservoirs. Microgels were introduced into the MF device from a syringe attached to the upstream reservoir using a three-way valve. The pressure difference, ΔP, applied along the microchannel was controlled by varying the difference in heights of the two reservoirs. The motion of the microgel was recorded with a high-speed camera (Canon EX-F1) and analyzed by a code written in MATLAB.

Supplementary Note 2. Determination of the flow rate of water through the microgel trapped at the entrance to the constriction

We employed fluorescence recovery after photobleaching (FRAP) in combination with confocal laser

scanning microscopy (CLSM) to measure the rate, Q, of water flow *through* a microgel trapped at the entrance to the constriction, as a function of applied pressure difference ΔP.

Before introducing a microgel into the MF channel, a 200 μm depth scan was performed at increments of 5 μm in the direction perpendicular to the imaging plane of the microchannel (z-direction). By using 3D reconstruction of the z-stack of CLSM images, we ensured that the microchannel had a close-to-circular cross-section (Supplementary Fig. S1a and b). FRAP experiments were performed at constant position, z = 0 μm, that is in, the middle plane of the circular microchannel (*see* Supplementary Fig. S1a), in order to avoid the attenuation of intensity that occurs in CLSM experiments when z-coordinate is varied. With the assumption of axisymmetry (explained in the derivation following the present section), the measurement of fluorescence intensity in the middle plane is sufficient to describe the fluorophore concentration throughout the geometry of the circular microchannel.

After introducing a microgel with an unperturbed diameter D_0 = 104 μm into a microchannel (d_c = 38 μm, α = 30°), a pressure difference not exceeding ΔP_{max} was applied to the MF system to confine the microgel in the tapered zone of the microchannel. An intense (100% power of 5.4 mW), 25 s laser pulse photobleached a rectangular (250 μm × 150 μm) region in the microchannel-at-large using the NIS Element software. To monitor the recovery of fluorescence, the program set the instrument to the attenuated beam (10% power) and a series of 20 images was recorded with 5 s intervals between image capturing. Photoemission intensity data, $I(x,r)$, were collected from the images (selected images shown in Supplementary Fig. S2a), where x is the coordinate in the flow direction measured from the left edge of the image, and r is the radial coordinate measured from the axis of the channel. The reference photoemission intensity, $I_0(x,r)$, was obtained from the image taken with the attenuated beam prior to the photobleaching event.

To determine the average velocity, U, of the fluid flowing through the microgel from the CLSM data, the normalized first axial moment M_1/M_0 of the cross-section-averaged fluorophore concentration, defined as

$$\frac{M_1}{M_0} = \frac{\int_0^l \int_0^{d/2} x[I(x,r) - I_0(x,r)] r dr dx}{\int_0^l \int_0^{d/2} [I(x,r) - I_0(x,r)] r dr dx}, \quad (1)$$

was calculated from the CLSM images taken at time intervals of 5 s. In equation (1), $I(x,r)$ is the fluorescence intensity distribution; x and r are the axial and radial positions coordinates; and l and d are the length and diameter of the microchannel in the image (*see* Supplementary Fig. S2a). The double integrals in equation (1) were calculated in MATLAB using the trapezoidal rule[2]. As shown in the next section, when the fluorescence intensity profile after bleaching is nearly a square pulse (that is, the 2D projection of the bleached region is nearly a rectangle), and the fluorophore distribution is axisymmetric, M_1/M_0 can be approximated by the following function of time

$$M_1/M_0 = X_0 + Ut. \quad (2)$$

The derivation of the equation (2) is given below. In equation (2), X_0 is the intercept of M_1/M_0 at $t = 0$. Since the radial dependence of fluorescence intensity can be measured either from the top half, or the bottom half of the CLSM image (the distribution is symmetric), the average value of M_1/M_0 (shown with black squares in Supplementary Fig. S2b) from the two halves was fit to equation (2); this is shown in Supplementary Fig. S2b. The average velocity, U, of the liquid flowing through the trapped microgel was obtained as the slope of the plot of M_1/M_0 vs. t, and found to be 0.35 ± 0.001 μm/s in the experiment shown in Supplementary Fig. S2. The flow rate, Q, of the liquid was determined as $Q = \pi (d/2)^2 U$ = 4019 ± 14 μm^3/s, where $d/2$ is the radius of the microchannel, measured from the CLSM images to be 60 μm.

The above procedure was implemented for a range of pressure differences varying from the value required to block the microchannel, up to ΔP_{max}. For each pressure difference, the FRAP experiment was repeated three times. Between repetitive FRAP measurements, a 5 min time interval was allowed for the system to reach a steady state. The variation in the average flow rate of the liquid through the microgel as a function of pressure difference is shown in Supplementary Fig. S3. For pressure difference below 2000 Pa, the flow rate of water was high, due to its leakage through the gaps between microchannel walls and the microgel. The estimation of this effect is given in Supplementary Note 4.

Supplementary Note 3. Interpretation of photobleaching data

In the derivation of equation (2), we followed the work of Aris[3]. Consider the pressure-driven flow of a Newtonian fluid through a circular tube. The flow occurs in the x direction, and r and θ are the cross-sectional coordinates in the polar form. The governing equation for any disturbance, $\Delta\rho$, in the unbleached fluorophore concentration distribution is the time-dependent convective diffusion equation

$$\frac{\partial \Delta\rho}{\partial t} + u\frac{\partial \Delta\rho}{\partial x} = D_f \nabla_2^2 \Delta\rho + D_f \frac{\partial^2 \Delta\rho}{\partial x^2}. \quad \textbf{(3)}$$

Here, D_f is the diffusion coefficient of the fluorophore, x is the direction of flow, and u is the velocity in the flow direction

$$u = U\left(1 - \frac{r^2}{(d/2)^2}\right), \quad \textbf{(4)}$$

where U is the average velocity through the tube. The operator ∇_2 in equation (3) is the gradient in the cross-sectional coordinates r and θ. Note that $\Delta\rho$ is a disturbance in the concentration of the fluorophore. For example, if the initial concentration of the fluorophore is ρ_0 and the concentration at a later time is ρ, then the disturbance, $\Delta\rho$, in the concentration is defined as $\Delta\rho = \rho - \rho_0$. In the experiment, the disturbance is introduced in the form of a rectangular region by photobleaching, and is, therefore, non-zero only in that region. As shown in the derivation that begins in the next paragraph, the axial velocity of the center of mass of the concentration disturbance is equal to the average velocity of the fluid. This result is straightforward to understand, when diffusion is absent, but is valid even when there is diffusion-induced radial and axial smearing of the concentration, which can be explained as follows. Since the initial fluorescence intensity distribution produced by photobleaching is rectangular, it is symmetric about the center of mass at t = 0. As time proceeds, convection advances to the two edges of the rectangle to the right *via* the parabolic velocity profile, thereby establishing concentration gradients in the radial direction

(*see* Fig. S3a). At the left edge, there is radial diffusion of the fluorophore from the center towards the walls, along with axial diffusion in the flow direction. Conversely, at the right edge, the fluorophore diffuses radially from the walls to the center, and axially - in the direction opposite to the flow. The key is that the diffusional distortions produced at the two edges complement each other exactly, their axial moments sum to zero, and do not contribute to the overall axial motion of the center of mass of the distribution. This is demonstrated more rigorously in the derivation below.

Multiplying equation (3) by x^n, and integrating in x from 0 to l yields

$$\frac{\partial C_n}{\partial t} = nuC_{n-1} + D_\mathrm{f}\left[\nabla_2^2 C_n + n(n-1)C_{n-2}\right]. \quad \mathbf{(5)}$$

Here, C_n is the nth axial moment of Δρ, defined as

$$C_n = \int_0^l \Delta\rho \; x^n dx. \quad \mathbf{(6)}$$

For example, C_0, the zeroth axial moment of Δρ, is

$$C_0 = \int_0^l \Delta\rho \; dx. \quad \mathbf{(7)}$$

In deriving equation (5), we have assumed that the concentration disturbance at the two ends of the tube, $x = 0$ and $x = l$, is negligible. This is a valid assumption provided that the photobleached region far from the edges of the tube throughout the duration of the experiment. This condition was maintained in FRAP experiments. By integrating equation (5) over the cross-section of the conduit, we obtain

$$\frac{dM_n}{dt} = n\iint_S uC_{n-1}dydz + D_\mathrm{f}n(n-1)M_{n-2}, \quad \mathbf{(8)}$$

where M_n is the cross-section-integrated value of C_n,

$$M_n = \iint_S C_n dA, \quad \mathbf{(9)}$$

where dA is an elemental cross-sectional area.

For $n = 0$, equation (8) becomes

$$\frac{dM_0}{dt} = 0,$$

implying that

$$M_0 = \iint_S C_0 dA = \text{constant, independent of time.} \quad \textbf{(10)}$$

Examination of equation (5) for n = 0 gives the governing equation for C_0 (equation (7))

$$\frac{\partial C_0}{\partial t} = D_{\mathrm{f}} \nabla_2^2 C_0, \quad \textbf{(11)}$$

with the boundary condition

$$\mathbf{n} \cdot \nabla_2 C_0 \big|_S = 0, \quad \textbf{(12)}$$

the integral constraint from equation (10),

$$\iint_S C_0 dA = M_0, \quad \textbf{(13)}$$

and the initial condition

$$C_0(r, \theta, 0) = \int_0^l \Delta\rho(x, r, \theta, 0) dx = \Gamma(r, \theta). \quad \textbf{(14)}$$

The function $\Gamma(r, \theta)$ is obtained by axially integrating the initial concentration distribution $\Delta\rho(x, r, \theta, 0)$.

The above problem has the following eigenfunction expansion solution

$$C_0(r, \theta, t) = \frac{M_0}{A} + \frac{M_0}{A} \sum_{j=0}^{\infty} \left[B_j \exp\left(-\mu_j \frac{D_{\mathrm{f}}\, t}{(d/2)^2} \right) \phi_j(r, \theta) \right]. \quad \textbf{(15)}$$

The functions ϕ_j and μ_j are the eigenfunctions and eigenvalues, respectively, of the following linear problem

$$\nabla_2^2 \phi_j = -\mu_i \phi_j, \quad \textbf{(16)}$$

with the boundary condition

$$\mathbf{n} \cdot \nabla_2 \phi_j \big|_S = 0. \quad \textbf{(17)}$$

The constants B_j are derived from the initial condition for C_0 in equation (14) using biorthogonality

condition,

$$\iint_S \phi_j \phi_i dA = \begin{cases} 0 & , \text{ for } i \neq j \\ \iint_S \phi_i^2 dA & , \text{ for } i = j \end{cases}, \tag{18}$$

as

$$B_j = \begin{cases} \frac{A}{M_0} \frac{\iint_S \Gamma \phi_j dA}{\iint_S \phi_j^2 dA} & , \text{ for } j \neq 0 \\ -1 + \frac{1}{M_0} \iint_S \Gamma dA & , \text{ for } j = 0 \end{cases}. \tag{19}$$

Note that Γ is the axial integral of the initial concentration distribution (*see* equation (14)). Since $\iint_S \Gamma dA = M_0$, the constant B_0 = zero. In addition, for the special case of a square pulse in the initial concentration profile that is imposed in the experiment, $B_j = 0$ for all $j \neq 0$, as well, implying that $B_j = 0$ for all j. Note that for a square pulse, Γ is independent of the cross-sectional coordinates. The null eigen-function of the operator in equation (16) (i.e. $\mu_0 = 0$) is $\phi_0 = 1$, and satisfies the following biorthogonality condition

$$\iint_S \phi_1 \phi_j dA = \iint_S \phi_j dA = 0 \text{ for } j \neq 0. \tag{20}$$

Equation (20), combined with equation (19), gives $B_j = 0$ for $j \neq 0$.

Since $B_j = 0$ for j, the solution for C_0 reduces from equation (15) to:

$$C_0\left(r,\theta,t\right) = \frac{M_0}{A}. \tag{21}$$

Expressing equation (8) for $n = 1$ yields

$$\frac{dM_1}{dt} = \iint_S u C_0 dA. \tag{22}$$

Substituting for C_0 from equation (21) gives

$$\frac{dM_1}{dt} = \iint_S u \frac{M_0}{A} dA = M_0 \left(\frac{1}{A} \iint_S u dA \right) = M_0 U. \tag{23}$$

Dividing the above equation by M_0, we obtain

$$\frac{d\left(M_1/M_0\right)}{dt}=U. \quad \textbf{(24)}$$

Integration w.r.t. time results in the equation

$$M_1/M_0 = X_0 + Ut. \quad \textbf{(25)}$$

Thus, by fitting the temporal variation in the ratio M_1/M_0 to the linear equation (15), we obtain U as the slope. For a square pulse at $t = 0$, the spatial distribution of the fluorophore concentration is axisymmetric at every instant, which enables the description of the cross-sectional variations of the fluorophore concentration with only the radial coordinate, r. The elemental area, dA, for the double integral is $2\pi r\ dr$. With this simplification, the definition of M_1/M_0 given in equation (1) can be used.

In practice, the assumptions of axisymmetry and square pulse are not exact. To be more accurate, all the eigenmodes in the solution of C_0 in equation (15) (except the $j = 0$ mode) have to be considered, which would result in the following expression for M_1/M_0:

$$\frac{M_1}{M_0} = X_0 + Ut + \frac{U(d/2)^2}{D_\mathrm{f}}\sum_{j=1}^{\infty} b_j \left[1 - \exp\left(-\mu_j \frac{D_\mathrm{f}\, t}{(d/2)^2}\right)\right]. \quad \textbf{(26)}$$

A linear regression of equation (26) will provide the value of U. However, the calculation of M_1/M_0 would require the fluorescence intensities throughout the cross-section to be measured as a function of r and θ, which is not possible in our experimental setup due to the time required for a complete cross-sectional confocal scan, and the dependence of the intensity on the z-position.

Supplementary Note 4. Estimation of the effect of leakage of water through the gaps between the microgel and microchannel walls

At small pressure difference ΔP applied to the system, leakage of water may occur due to the non-conformal contact between the microgel trapped at the entrance to the constriction and microchannel walls. If the hydraulic resistance to flow through these gaps is smaller than that for the flow through the gel, the total water flow will be dominated by the leakage through the gaps, thus biasing the results. In

this Supplementary Note, we estimate the effect of applied pressure difference, ΔP, on the leakage flow and show that significant leakage occurs only for $\Delta P < E_0$.

Since the geometry of the non-conformal contact between the microgel and the wall is not known *a priori*, our estimation was based on the assumed gap geometry. First, we verified that a small mismatch between the spherical microgel shape and the elliptical channel cross-section cannot explain the experimentally observed pressure difference required to close the gaps. Thus we concluded that the mismatch between the channel geometry and the microgel shape should have different characteristics.

We considered the gaps between the microgel and the channel wall to be due to surface roughness (Supplementary Fig. S4) and estimated the effective spacing g of surface irregularities on microchannel wall, as well as the pressure difference, ΔP_c, required to close the gaps between the microgel and the channel surface.

From Hertzian theory of an elastic contact between two spheres[4], we estimate the diameter, a, of the contact between the spheres and applied force, $\mathcal{F}$, as functions of strain (h):

$$a \approx (D_c\, h)^{1/2} \quad \text{and} \quad \mathcal{F} \approx E^* (D_c\, h^3)^{1/2}. \qquad \mathbf{(27)}$$

Here, $D_c/2$ is the effective radius at the contact surface, $D_c = (1/g + 1/D_0)^{-1}$, and E^* is related to the Young's modulus, E_0, and Poisson's ratio, σ, of the microgel as $E^* = 4E_0/[3(1-\sigma^2)]$, assuming rigid microchannel walls. Note that $D_c \approx g$ for $g \ll D_0$ and $E^* \approx E_0$ for $\sigma \approx 1$.

For the geometry shown in Supplementary Figure S4c, L is the axial length of region of contacts, and LD_0 is proportional to the area of contact spots. Assuming the number of contacts $n \approx LD_0/g^2$, the total force imposed on the microchannel wall is $n\mathcal{F} \approx LD_0\mathcal{F}/g^2$. This force is supported by the pressure difference ΔP. Since the radial wall pressure is proportional to ΔP, the total contact force is $n\mathcal{F} \approx LD_0\mathcal{F}/g^2 \approx \Delta P\, LD_0$, and thus, the force acting on a single contact surface is $\mathcal{F} \approx \Delta P\, g^2$. Substitution of the force $\mathcal{F}$ from equation (27) with $D_c \approx g$ and $E^* \approx E_0$, gives

$$\Delta P/E_0 \approx \mathcal{F}/(E^* g^2) \approx (h/g)^{3/2}. \qquad \mathbf{(28)}$$

The gaps are closed when $h \approx a \approx g$. Thus, from equation (28), the pressure difference ΔP_c required to

close the leaking gaps is

$$\Delta P_c \approx E_0, \qquad \textbf{(29)},$$

which agrees with experimental results (*see* Supplementary Fig. S3a). For $\Delta P > \Delta P_c$, water flows only through the microgel without leakage.

The flow rate of water through the gaps is predicted as $Q_{gap} = \Delta P/R_{gap}$, where

$$R_{gap} \approx [g/D_0]\ \eta\ L\ (g - h)^{-3}\ (g - a)^{-1} \qquad \textbf{(30)}$$

is the hydraulic resistance of the gaps[5]. The prefactor in square brackets is due to water leakage occurring simultaneously through D_0/g gaps. The axial length of the contact region can be determined from the Hertzian theory for an elastic contact between two cylinders as[4] $L \approx (E^*\mathcal{F})^{1/2}$, where the force on walls is, $\mathcal{F} \approx D_0^2\Delta P$. Hence,

$$L \approx D_0\ (\Delta P/\Delta P_c)^{1/2}. \qquad \textbf{(31)}$$

Combining equations (27-31), we obtain

$$\frac{Q_{\text{gap}}}{E_0/\eta} \approx g^3 \left(\frac{\Delta P}{\Delta P_c}\right)^{1/2} \left(1 - \left(\frac{\Delta P}{\Delta P_c}\right)^{2/3}\right)^3 \left(1 - \left(\frac{\Delta P}{\Delta P_c}\right)^{1/3}\right), \qquad \textbf{(32)}$$

which is plotted in Supplementary Fig. S5, as blue curve.

Equation (32) predicts an increase of water flux, $Q_{\text{gap}} \sim \Delta P^{1/2}$, for a very small pressure difference, $\Delta P \ll \Delta P_c$. This regime is followed by the shrinkage of gaps and decrease in Q_{gap}, as observed in experiments (*see* left-most three data points in Supplementary Fig. S3a, which correspond to the leakage-dominated regime). At $\Delta P = \Delta P_c$, gaps close and Q_{gap} vanishes (*see* equation (32)). At $\Delta P > \Delta P_c$, water flows only through the microgel without leakage. In the last section of Supplementary Note 5, for low ΔP, we estimated this flow through the microgel to be $Q_{gel} = \Delta P/R_0$, with constant hydraulic resistance $R_0 = 1$ Pa·s/μm^3 (*see* equation (75) and red lines in Fig. 3b and Supplementary Fig. S5). Hence, the total flow rate is given by $Q = Q_{gap} + Q_{gel}$.

We estimated the size, g, of gaps and the pressure, ΔP_c, required to close the gaps by fitting the theoretical prediction for $Q(\Delta P)$ to the experimental Q-ΔP data in the leakage dominated regime and the

following linear response regime. Such a fit resulted in the best fitting parameters $g = 0.38 \pm 0.02$ μm and $\Delta P_c/E_0 = 1.9 \pm 0.2$, which agreed with experimental data (Supplementary Fig. S5). We note that the reported surface roughness of PDMS surface is in the submicrometer range[6].

We therefore conclude that at pressure differences $\Delta P < E_0$, a large number of small-roughness elements with submicrometer size can provide leakage of water comparable to the flow within the gel. The size of imperfections (on the order $g \approx 0.1$ μm), the value of ΔP required for closing the gaps (on the order $\Delta P_c \approx E_0$), and the decrease in flow rate Q with increasing ΔP in the leakage-dominated regime (equation (32) and Supplementary Fig. S5), all agree with the experimental results. Furthermore, we estimate the hydraulic resistance of gaps as $R_{gap} \approx 0.1$ - 1 Pa·s/μm^3 from the left-most three data points in Supplementary Fig. S3a, while the hydraulic resistance of microgel $R_0 = 1$ Pa·s /μm^3 is observed for $\Delta P > E_0$ in the linear response regime (equation (75) and red lines in Fig. 3b and Supplementary Fig. S5). Hence, by comparing the two flow resistances, we conclude that for $\Delta P > E_0$ the flow of water occurs primarily through the microgel. We should also note that with a cubic $(g - h)^3$ scaling, the leakage flow is highly sensitive to the height of the gaps. Thus, once the gaps are smaller than a critical height (corresponding to equal leakage and microgel flow rates), the leakage flow rapidly diminishes.

Supplementary Note 5. Theoretical predictions of the behavior of microgels in confinement

1. Deformation free energy density

Consider a microgel prepared in a good solvent at the monomer number density c_i that is swollen to an equilibrium concentration c_0 and volume V_0. The equilibrium swelling corresponds to the deformation factors $\lambda_0 = (c_i/c_0)^{1/3}$ in all three dimensions of the microgel. In the microchannel, the microgel is locally deformed by additional factors $\lambda_x = \lambda_{||}$ in the axial direction and $\lambda_y = \lambda_z = \lambda_\perp$ in the transverse directions to a new number density c of monomers

$$c = c_0/\left(\lambda_{||}\,\lambda_{\perp}^2\right). \quad \textbf{(33)}$$

The free energy of the microgel is the sum of elastic and osmotic components[7]. We use the predictions of scaling theory for these two contributions to the microgel free energy. The osmotic pressure π of the microgel in a good solvent increases proportionally to the 9/4 power of its concentration[8,9]

$$\pi \approx (kT/b^3)(cb^3)^{9/4}, \quad \textbf{(34)}$$

where b is the size of the monomer. The osmotic part f_{os} of the free energy per polymer chain between network junctions is proportional to the free energy density (~ π) divided by the number of chains per unit volume (c/N), where N is the chain degree of polymerization. The osmotic contribution to the free energy per chain scales with polymer concentration c as

$$f_{os} \approx kTN(cb^3)^{5/4}. \quad \textbf{(35)}$$

We assume that individual network chains between crosslinks deform affinely, that is, proportionally to the dimensions of the entire microgel. In this case, the dimensions of the chain in the deformed microgel is $\bar{R}_{\|} = \lambda_{\|}\bar{R}_0 = \lambda_{\|}\lambda_0\bar{R}_{\mathrm{i}}$ in the axial direction and $\bar{R}_{\perp} = \lambda_{\perp}\bar{R}_0 = \lambda_{\perp}\lambda_0\bar{R}_{\mathrm{i}}$ in the transverse direction, where $\bar{R}_0$ and $\bar{R}_{\mathrm{i}}$ are the chain sizes in the fully swollen state (at $c = c_0$) and in the state at which the gel was formed (at $c = c_{\mathrm{i}}$), respectively. The elastic free energy per chain scales as

$$f_{\mathrm{el}} \approx kT\sum_j \left(\frac{\bar{R}_j}{\bar{R}_{\mathrm{f}}}\right)^2 \approx kT\sum_j \left(\lambda_j\lambda_0\frac{\bar{R}_{\mathrm{i}}}{\bar{R}_{\mathrm{f}}}\right)^2, \quad \textbf{(36)}$$

where $\bar{R}_{\mathrm{f}}$ is the amplitude of fluctuations of the chain in the deformed microgel state, and the sum is over independent $j = \{x,y,z\}$ directions. Since the mean-square amplitude of chain fluctuations is proportional to the mean-square polymer size at semi-dilute good solvent conditions and scales as[8,9] $\bar{R}_{\mathrm{f}}^2 \sim c^{-1/4}$, while the mean-square chain size in the preparation conditions scales as $\bar{R}_{\mathrm{i}}^2 \sim c_{\mathrm{i}}^{-1/4}$, the elastic free energy per chain is

$$f_{\mathrm{el}} \approx kT\left(\frac{c}{c_{\mathrm{i}}}\right)^{1/4}\lambda_0^2\sum_j\lambda_j^2 \approx kT\left(\frac{c}{c_0}\right)^{1/4}\left(\frac{c_{\mathrm{i}}}{c_0}\right)^{5/12}\sum_j\lambda_j^2. \quad \textbf{(37)}$$

At the equilibrium swelling ($c = c_0$) in the absence of additional deformations, $\sum_j\lambda_j^2 = 3$, the total

free energy of the microgel (*see* equations (35) and (37))

$$f = f_{\text{os}} + f_{\text{el}} \approx kT\left[N(c_0 b^3)^{5/4} + (c_{\text{i}}/c_0)^{5/12}\right] \qquad \text{at } c = c_0 \tag{38}$$

is minimized ($\partial f/\partial c_0 = 0$) at the concentration[9]

$$c_0 \approx \frac{(c_{\text{i}} b^3)^{1/4}}{b^3 N^{3/5}}. \tag{39}$$

Using this relation between the concentrations at the preparation conditions and the swelling equilibrium $(c_{\text{i}} / c_0)^{5/12} = N(cb^3)^{5/4}$, as well as the fact that the ratio of polymer concentrations is $c/c_0 = (\lambda_{\parallel}\lambda_{\perp}^2)^{-1}$ (*see* equation (33)), one can write the total free energy per chain in the deformed state within the scaling approximation (equations (35) and (37)) as[10]

$$f \approx kTN(c_0 b^3)^{5/4}\left[\left(\lambda_{\parallel}\lambda_{\perp}^2\right)^{-5/4} + \left(\lambda_{\parallel}\lambda_{\perp}^2\right)^{-1/4}\left(2\lambda_{\perp}^2 + \lambda_{\parallel}^2\right)\right], \tag{40}$$

where the sum of squares of the deformation factors in equation (37) is $\sum_j \lambda_j^2 = 2\lambda_{\perp}^2 + \lambda_{\parallel}^2$. Note that the free energy per unit volume of the fully swollen microgel fc_0/N normalized by the modulus of the fully swollen gel,

$$E_0 \approx \frac{kT}{b^3}(c_0 b^3)^{9/4}, \tag{41}$$

is universal and depends only on the dimensionless deformation factors

$$\frac{\bar{f}}{E_0} = \frac{fc_0}{NE_0} = \frac{11}{50}\left[\left(\lambda_{\parallel}\lambda_{\perp}^2\right)^{-5/4} + \left(\lambda_{\parallel}\lambda_{\perp}^2\right)^{-1/4}\left(2\lambda_{\perp}^2 + \lambda_{\parallel}^2\right)\right]. \tag{42}$$

This is the reason why normalizing pressure difference ΔP by the modulus E_0 of the swollen microgels generated universal dependences in Figs. 4b and 5b. Note that we have chosen the numerical coefficient in equation (42) to be 11/50, to assure that E_0 is the Young's modulus.

Below we present the scaling relations between (*i*) the position of the microgel and the applied pressure difference, (*ii*) the reduction in microgel volume and the degree of microgel confinement, and (*iii*) the translocation pressure difference and the degree of confinement.

2. Deformation of a spherical microgel

In the steady state the size and shape of the spherical microgel under compression are determined by the condition of force balance, and below we calculate forces acting on the microgel. The walls of the microchannel impose a non-uniform deformation on the spherical microgel. Below, we describe the calculation of the deformation of a spherical microgel, neglecting the bending effects and the deformation of the caps (Supplementary Fig. S6). Such an approach is more applicable for the microgel confinement in a microchannel with small entrance angles α.

The total free energy of deformation, F, is an integral over the whole volume of the undeformed microgel

$$F = \int_{-D_0/2}^{D_0/2} \bar{f}[\lambda_{\parallel}(x_0), \lambda_{\perp}(x_0)] A_0(x_0)\, dx_0, \quad \textbf{(43)}$$

where x_0 denotes the axial coordinate in the undeformed microgel state (with the origin at the center of the microgel) and $A_0(x_0) = \frac{\pi}{4}(D_0^2 - 4x_0^2)$ is the circular cross-sectional area of the undeformed microgel at x_0 (Supplementary Fig. S6).

Consider infinitesimal disk-shaped slices of the undeformed microgel. We assume that when the microgel is confined in the microchannel, each disk of diameter $\sqrt{D_0^2 - 4x_0^2}$ and thickness dx_0 at the axial position x_0 in the undeformed microgel state uniformly transforms into another disk of diameter $D[x(x_0)]$ and thickness dx at the axial position $x(x_0)$. Thus we neglect the bending of the disks upon deformation. The shape of the microchannel (Supplementary Fig. S6) is the cylindrical constriction with constant diameter $D(x \geq 0) = d_c$ for the positive coordinate x, and the tapered truncated cone region $D(x \leq 0) = d_c - 2x \tan\alpha$, for the negative coordinate x. The radial and axial deformation ratios are thus

$$\lambda_{\perp}(x_0) = \frac{D[x(x_0)]}{\sqrt{D_0^2 - 4x_0^2}} \quad \text{and} \quad \lambda_{\parallel}(x_0) = \frac{dx}{dx_0}. \quad \textbf{(44)}$$

We minimize the total free energy *via* standard variational method[4]. In addition to the large deformation described in equation (44), we define an infinitesimal deformation by $x \rightarrow x_u = x + u(x)$. This

variation in the axial coordinates of the disks, $\delta x = x_u - x = u(x)$, yields the variations of the deformation factors

$$\lambda_\perp(x_0) \to \lambda_{\perp u}(x_0) = \lambda_\perp(x_0) + \frac{D'(x)}{D(x)} \lambda_\perp(x_0)\, u(x_0) \quad \Rightarrow \quad \delta\lambda_\perp = \frac{D'(x)}{D(x)} \lambda_\perp(x_0)\, u(x_0)$$

$$\lambda_\parallel(x_0) \to \lambda_{\parallel u}(x_0) = \lambda_\parallel(x_0) + u'(x_0) \quad \Rightarrow \quad \delta\lambda_\parallel = u'(x_0) \qquad \textbf{(45)}$$

and the variation in total free energy

$$\delta F = \int \bar{f}[\lambda_{\perp u}(x_0), \lambda_{\parallel u}(x_0)] A_0(x_0) dx_0 - \int \bar{f}[\lambda_\perp(x_0), \lambda_\parallel(x_0)] A_0(x_0) d\mathrm{x}_0 \,. \qquad \textbf{(46)}$$

Substituting $\lambda_{\perp u}$ and $\lambda_{\parallel u}$ from equation (45) into this δF, expanding it in series up to first order in u and u', and using integration by parts, we obtain

$$\delta F = \left[\frac{\partial \bar{f}(\lambda_\perp, \lambda_\parallel)}{\partial \lambda_\parallel} A_0(x_0)\, u(x_0)\right]\Bigg|_{x'_{0\mathrm{b}}}^{x'_{0\mathrm{f}}}$$

$$- \int \left[\frac{d}{dx_0}\left(\frac{\partial \bar{f}(\lambda_\perp, \lambda_\parallel)}{\partial \lambda_\parallel} A_0(x_0)\right) - \frac{D'(x)}{D(x)} \lambda_\perp(x_0) \frac{\partial \bar{f}(\lambda_\perp, \lambda_\parallel)}{\partial \lambda_\perp} A_0(x_0)\right] u(x_0) dx_0 \,. \qquad \textbf{(47)}$$

Using $\lambda_\parallel = dx/dx_0$, $\lambda_\perp^2 = A(x)/A_0[x(x_0)]$, and defining local stress in j-direction

$$\sigma_{jj} = [\lambda_j/(\lambda_\perp^2\, \lambda_{||})]\, (\partial \bar{f}/\partial \lambda_j), \qquad \textbf{(48)}$$

we simplify equation (47) as

$$\delta F = [\sigma_{xx}(x)\, A(x)\, u(x)]\big|_{x'_\mathrm{b}}^{x'_\mathrm{f}} - \int_{x'_\mathrm{b}}^{x'_\mathrm{f}} \left[\sigma'_{xx}(x) + \frac{D'(x)}{D(x)}(2\sigma_{xx}(x) - \sigma_{rr}(x))\right] A(x)\, u(x)\, dx. \quad \textbf{(49)}$$

This extra free energy is stored in the microgel due to the conversion of the work δW

$$\delta W = -\int_{x'_\mathrm{b}}^{x'_\mathrm{f}} \mathcal{F}_x^{\mathrm{int}}(x)\, A(x)\, u(x)\; dx \qquad \textbf{(50)}$$

performed on it. Here, $\mathcal{F}_x^{\mathrm{int}}(x)$ is the body force (force in x-direction per unit volume) acting on disks. Assumptions of the “undeformed caps” and “no bending of disks” imply zero stress at the back and front boundaries of the deformed part of the microgel

$$\sigma_{xx}(x'_\mathrm{b}) = \sigma_{xx}(x'_\mathrm{f}) = 0. \qquad \textbf{(51)}$$

Note that $\sigma_{xx}(x'_{\mathrm{b\backslash f}}) = 0$ requires $\lambda_\perp(x'_{\mathrm{b\backslash f}}) = \lambda_\parallel(x'_{\mathrm{b\backslash f}}) = 1$ at the front and back boundaries of the deformed section of the microgel. Inside the deformed part of the microgel $\mathcal{F}_x^{\mathrm{int}}(x) = -P'(x)$ results in

$$\sigma'_{xx}(x) + \frac{D'(x)}{D(x)}(2\sigma_{xx}(x) - \sigma_{rr}(x)) + P'(x) = 0\ , \qquad \textbf{(52)}$$

where $P'(x)$ is the water pressure gradient. For a steady state, these conditions correspond to the force balance at the back and front boundaries of the deformed part of the microgel (equation (51)) and inside the deformed section of the microgel (equation (52)), respectively.

3. Pressure gradient along the microgel

In order to relate the water pressure gradient $P'(x) = dP/dx$ to the deformation along the microgel, we modeled each elementary vertical slice of a microgel (a disk of diameter $D(x)$ and thickness dx) at the axial position x by a densely packed set of parallel "*pipes*" with a diameter $\xi(x)$ (the correlation length), which scales with polymer concentration as $\xi = \xi_0\ (c/c_0)^{-3/4}$; thus, $\xi = \xi_0\ (\lambda_\perp^2\ \lambda_{||})^{3/4}$ (*see* equation (33)). The pressure difference dP across the pipe is $dP = q\ dR_\xi$, where dR_ξ is the hydraulic resistance of each pipe and q is the volumetric flow rate of water through the pipe (*e.g.*, in units of $\mu m^3/s$). Each disk of diameter $D(x)$ and length dx accommodates $m \approx (D/\xi)^2$ pipes, and the total resistivity of a disk containing m-pipes acting in parallel is $dR = dR_\xi/m$. The flow rate through the disk is $Q = mq$.

The flow of liquid inside the pipes is assumed to be laminar and described by the Poiseuille law, which gives the resistance of a pipe $dR_\xi = (128\eta\ dx)/(\pi\ \xi^4)$, where η is the viscosity of the liquid[5]. Thus the resistivity of a disk at position x is

$$dR\ =\ (128\eta\ dx)/[\pi D^2(x)\ \xi^2(x)] \qquad \textbf{(53)}$$

and the pressure gradient at position x is $dP/dx = (128\ \eta\ Q)/(\pi\ D^2(x)\ \xi^2(x))$. Note that Eq. (53) can be treated as Darcy's law for the flow through a porous medium with non-uniform permeability along the microgel as $\kappa = \xi^2/32$, since Darcy's law predicts[5] $dR = (4\eta dx)/(\pi D^2\kappa)$. By substituting the correlation length $\xi = \xi_0\ (\lambda_\perp^2\ \lambda_{||})^{3/4}$ for good solvent conditions, we obtain

$$dP/dx\ =\ [(128\ \eta\ Q)/(\pi\ \xi_0^2)]D^{-2}(x)\ [\lambda_\perp^2(x)\ \lambda_{||}(x)]^{-3/2}, \qquad \textbf{(54)},$$

which must be balanced by the body force inside the deformed microgel, as was given in equation (52). By using the geometry of the setup and the free energy density of the microgel (given in equation (42)) in the calculation of the stress components σ_{jj}, we obtain

$$\frac{\lambda_{\parallel}'(x)}{\lambda_{\parallel}(x)} = \frac{-2}{D(x)}\left[\frac{D'(x)[45-30\lambda_{\perp}^{4}(x)\lambda_{\parallel}(x)-3\lambda_{\perp}^{2}(x)\lambda_{\parallel}^{3}(x)]+G(x)[45+2\lambda_{\perp}^{4}(x)\lambda_{\parallel}(x)-35\lambda_{\perp}^{2}(x)\lambda_{\parallel}^{3}(x)]-\frac{\ell_0}{D(x)}[\lambda_{\perp}^{2}(x)\lambda_{\parallel}(x)]^{3/4}}{45+10\lambda_{\perp}^{4}(x)\lambda_{\parallel}(x)+21\lambda_{\perp}^{2}(x)\lambda_{\parallel}^{3}(x)}\right], \tag{55}$$

where $\lambda_{\parallel}' = d\lambda_{\parallel}/dx$, and we have used an abbreviation for the characteristic flow rate (in units of length), $\ell_0 = (51200\ \eta\ Q)/(11\pi\ \xi_0^2\ E_0)$. Note that $\ell_0 \sim Q$ appears in $\lambda_{\parallel}'/\lambda_{\parallel}$ in the above equation since flow deforms the microgel in the axial direction. In addition to equation (55), the definition of $\lambda_{\perp}$ given in equation (44), leads to

$$\frac{\lambda_{\perp}'(x)}{\lambda_{\perp}(x)} = \frac{D'(x)+G(x)}{D(x)}, \tag{56}$$

where $\lambda_{\perp}' = d\lambda_{\perp}/dx$. In equations (55) and (56), we used a function

$$G(x) = \mp \frac{2\lambda_{\perp}^{2}(x)}{\lambda_{\parallel}(x)}\sqrt{\left|\left(\frac{D_0}{D(x)}\right)^{2} - \frac{1}{\lambda_{\perp}^{2}(x)}\right|}. \tag{57}$$

Here, the positive and negative signs are for the two sides of the unperturbed microgel, $x_0 < 0$ and $x_0 > 0$ respectively, as the unperturbed diameter of a disk increases or decreases with x_0, depending on which side of the gel it is.

4. Results of numerical simulations

The coupled differential equations (55) and (56), corresponding to the force balance inside the microgel and the shape of the microgel imposed by the confinement are numerically integrated *via* 4th order Runge-Kutta algorithm[2] for the geometrical parameters relevant to the geometry of the microchannel. The assumption of the undeformed microgel caps corresponds to the boundary conditions of $\lambda_{\perp}(x'_{b\backslash f}) = \lambda_{\parallel}(x'_{b\backslash f})$

= 1 at the back and front boundaries of the microgel portion in contact with the microchannel walls. Note that the definition of $\lambda_{\|}$ in equation (44) implies $dx_0 = dx/\lambda_{\|}$, which can be used to transform the integrals over x_0 in the undeformed state into integrals over x in the deformed state. Thus we obtained the deformation ratios $\lambda_{\perp}(x|x'_{\mathrm{f}})$, $\lambda_{\|}(x|x'_{\mathrm{f}})$ and the normalized axial stress $\sigma_{xx}(x|x'_{\mathrm{f}})/E_0$ inside the microgel at the axial position x for a particular front boundary position x'_{f}. The flow rate Q was adjusted by a bisection algorithm to obtain the undeformed back boundary.

In order to determine the position x_{c}, where the function $G(x)$ (introduced in equations (55) and (56)) changes its sign, we also performed an axial length integration

$$S(x) = \int_{x_0}^{x'_{0\mathrm{f}}} d\tilde{x}_0 = \int_{x}^{x'_{\mathrm{f}}} \lambda_{\|}^{-1}(\tilde{x}) d\tilde{x}. \quad \textbf{(58)}$$

From the geometry defined in Supplementary Fig. S6, the center of the undeformed microgel ($x_0 = 0$) corresponds to $S(x_{\mathrm{c}}) = \frac{1}{2}\sqrt{D_0^2 - D_{\mathrm{f}}^2}$.

In Supplementary Fig. S7, we illustrate the numerical results of our theoretical approach described above. Supplementary Figs. S7a and b show the numerical results for profiles of deformation ratios ($\lambda_{\perp}$, $\lambda_{\|}$) and normalized concentration (c/c_0) along the deformed microgels at different positions inside the microchannel. At translocation instability point (at $\Delta P = \Delta P_{\mathrm{max}}$, grey lines), the largest microgel compression is at the constriction entrance ($x = 0$). Based on Supplementary Figure S7b, the respective increase in polymer concentration is in the range $1 \leq c/c_0 \leq 3.3$. Recalling that $\kappa/\kappa_0 = (\xi/\xi_0)^2 = (c/c_0)^{-3/2}$, we conclude that the ratio of permeabilities κ/κ_0 varies in the range $1 \geq \kappa/\kappa_0 \geq 0.2$. Here κ_0 is the permeability of the undeformed microgel, which was estimated to be approximately 7 nm² from Eq. (75). For the highest increase in polymer concentration of $c/c_0 \approx 3.3$, the largest reduction in permeability by the factor of 5 (or the smallest ratio $\kappa/\kappa_0 \approx 0.2$) was obtained. In Supplementary Fig. S7c, we plot the local volume change factor ($\lambda_{\perp}^2 \lambda_{\|}$) as a function of local radial deformation factor ($\lambda_{\perp}$) using the data in Supplementary Fig. S7a. The relation $\lambda_{\perp}^2 \lambda_{\|} \sim \lambda_{\perp}^{4/3}$ (obtained for no external axial stress from equation (63), dashed line) is in good agreement with the numerical data. In Supplementary Fig. S7d, we plot the

calculated flow rate as a function of pressure difference and compare the numerical data with scaling predictions.

Finally, Supplementary Fig. S7e shows the numerical result for the normalized front tip position of the microgel in the constriction, $x_f/d_c > 0$, as a function of normalized pressure difference, $\Delta P/E_0$, compared to experimental data. Fitting a function in the power-law form, $x_f/d_c \approx (\Delta P/E_0)^a$, to the numerical data leads to $a = 2.3 \pm 0.1$ for a broad range of pressure differences. The universal scaling relation

$$x_{\mathrm{f}}/d_{\mathrm{c}} \approx (\Delta P/E_0)^{2.3} \qquad \text{for} \quad x_{\mathrm{f}} > 0, \qquad \textbf{(59)}$$

is also in agreement with the experimental results for the strongly constricted microgels. In the next section, we discuss scaling relations for volume reduction, V/V_0, and rescaled translocation pressure difference $\Delta P_{max}/E_0$.

5. Scaling predictions for a deformed microgel

In the Sections 2-4 above, we considered spherical microgels. Here, in order to obtain universal scaling predictions, we consider a microgel with a cylindrical undeformed shape and an equilibrium swollen diameter D_0 with its symmetry axis oriented in x-direction.

a. Volume reduction of a microgel in the constriction

If a microgel is completely inside the constriction at zero pressure difference $\Delta P = 0$ and no fluid flows through it ($Q = 0$), the microgel is unconstrained in the axial direction. The radial size of the microgel is equal to the diameter d_c of the constriction. Thus the radial deformation factor is constant $\lambda_\perp = d_c/D_0$. The longitudinal deformation factor $\lambda_\parallel$ of the microgel can be obtained by the minimization of the free energy per chain (equation (40)) or the free energy per unit volume of the fully swollen microgel (equation (42)) with respect to $\lambda_\parallel$ at constant $\lambda_\perp = d_c/D_0$. This minimization, $\partial\bar{f}/\partial\lambda_\parallel = 0$, corresponds to the balance of

elastic and osmotic stresses in the unconstrained axial direction

$$\frac{\partial \bar{f}}{\partial \lambda_{\parallel}} \approx \frac{11}{50} E_0 \left(-\frac{5}{4}\lambda_{\parallel}^{-9/4}\lambda_{\perp}^{-5/2} - \frac{1}{2}\lambda_{\parallel}^{-5/4}\lambda_{\perp}^{3/2} + \frac{7}{4}\lambda_{\parallel}^{3/4}\lambda_{\perp}^{-1/2}\right)$$

$$\approx -\frac{11E_0}{200}\lambda_{\parallel}^{-9/4}\lambda_{\perp}^{-5/2}\left(5 + 2\lambda_{\parallel}\lambda_{\perp}^{4} - 7\lambda_{\parallel}^{3}\lambda_{\perp}^{2}\right) = 0. \quad \textbf{(60)}$$

The solution of this cubic equation with $\lambda_{\perp} = d_c/D_0$ is[10]

$$\lambda_{||} = (5/7)^{1/3}(D_0/d_c)^{2/3}\left[(½ + v)^{1/3} + (½ - v)^{1/3}\right], \quad \textbf{(61)}$$

where

$$v = [1/4 - (8/4725)\,(D_0/d_c)^{-10}]^{1/2}. \quad \textbf{(62)}$$

Note that for large radial compressions $\lambda_{\perp} \ll 1$, the parameter $v \approx ½$ and

$$\lambda_{||} \sim \lambda_{\perp}^{-2/3} \qquad \text{for } \lambda_{\perp} \ll 1. \quad \textbf{(63)}$$

The relative change of volume upon microgel confinement in the constriction is

$$V/V_0 = \lambda_{\perp}^{2}\,\lambda_{||} = (5/7)^{1/3}(D_0/d_c)^{-4/3}\left[(½ + v)^{1/3} + (½ - v)^{1/3}\right], \quad \textbf{(64)}$$

which is equation (8) in the main text.

The above equation (64) (equation (8) of the main text) is obtained for a cylindrical shape of the undeformed microgel. The relations show a universal power-law dependence with an exponent –4/3 for geometry confinement, and are represented by the black curves in Fig. 6a and b of the main text. The experimental and theoretical results were in excellent agreement without any fitting parameters.

b. Dependence of the translocation pressure on the degree of microgel confinement

The dependence of ΔP_{max} on D_0/d_c can be obtained by integrating equation (52) along the microgel:

$$\Delta P = -\int_{x_b'}^{x_f'} dP = \int_{x_b'}^{x_f'} d\sigma_{xx} + \int_{x_b'}^{x_f'} \frac{dx}{\lambda_{\parallel}}\left[\frac{\lambda_{\parallel}}{D}\frac{dD}{dx}(2\sigma_{xx} - \sigma_{rr})\right]. \quad \textbf{(65)}$$

The first integral vanishes due to vanishing surface forces (equation (51)), and using $\int_{x_b'}^{x_f'} \frac{dx}{\lambda_{\parallel}} \approx D_0$, we estimate the second integral in equation (65) as

$$\Delta P_{\mathrm{max}} \approx \left[-D_0 \frac{\lambda_{\parallel}}{D} \frac{dD}{dx} (2\sigma_{xx} - \sigma_{rr}) \right] \Big|_{x_{\mathrm{m}}}. \qquad \textbf{(66)}$$

Here, the right-hand-side is taken at a certain point $x = x_{\mathrm{m}} = -\gamma(\alpha)\, d_{\mathrm{c}}$, near the maximum of the expression in square brackets in equation (65) inside the integration interval ($x_{\mathrm{b}}' \leq x \leq x_{\mathrm{f}}'$) at the translocation instability point ($\Delta P = \Delta P_{\mathrm{max}}$). For strong deformation ($\lambda_{\parallel} \approx \lambda_{\perp}^{-2/3} \gg \lambda_{\perp}$), from equations (42) and (48), we have $2\sigma_{xx} - \sigma_{rr} \approx E_0 \lambda_{\perp}^{-3}$, and substituting it into equation (66) along with the slope of the tapered zone, *i.e.*, $\mathrm{d}D/\mathrm{d}x = -2\tan\alpha$, we write

$$\frac{\Delta P_{\mathrm{max}}}{E_0} \approx \tan\alpha \left[\frac{D_0}{D} \lambda_{\perp}^{-11/3} \right] \Big|_{x_{\mathrm{m}}}. \qquad \textbf{(67)}$$

Here, making use of the cylindrical undeformed gel approximation, *i.e.*, $\lambda_{\perp} \approx D/D_0$, we obtain

$$\frac{\Delta P_{\mathrm{max}}}{E_0} \approx \tan\alpha\, [\lambda_{\perp}(x_{\mathrm{m}})]^{-14/3}. \qquad \textbf{(68)}$$

The radial deformation ratio at $x = x_{\mathrm{m}}$ is $\lambda_{\perp}(x_{\mathrm{m}}) = D(x_{\mathrm{m}})/D_0 = (d_{\mathrm{c}} - 2\tan\alpha\, x_{\mathrm{m}})/D_0 = (1 + 2\gamma(\alpha)\tan\alpha)\, (d_{\mathrm{c}}/D_0)$. Therefore, we have

$$\frac{\Delta P_{\mathrm{max}}}{E_0} = K(\alpha) \left(\frac{D_0}{d_{\mathrm{c}}} \right)^{14/3}, \qquad \textbf{(69)}$$

where we defined the angle-dependent prefactor

$$K(\alpha) \approx \tan\alpha\, (1 + 2\,\gamma(\alpha)\tan\alpha)^{-14/3}. \qquad \textbf{(70)}$$

The size dependence of translocation pressure difference, *i.e.*, the power-law $\Delta P_{\mathrm{max}} \approx (D_0/d_{\mathrm{c}})^{14/3}$, is universal with an exponent 14/3, independent of the shape of the tapering zone. However, the prefactor (amplitude) of this power-law, $K(\alpha)$, is not universal and depends on the geometry of the microchannel and the microgel. To this end, we consider simulation results to obtain the amplitude $K(\alpha)$ and compare them with experimental data.

In Supplementary Fig. S8a, we plot the simulation results for rescaled translocation pressure difference, $\Delta P_{\mathrm{max}}/E_0$, as a function of degree of confinement, D_0/d_{c}, for different tapering angles, α. We observe (*i*) persistent power-law behavior $\Delta P_{\mathrm{max}} \approx (D_0/d_{\mathrm{c}})^{14/3}$, and (*ii*) saturation of the angle-dependent amplitude for large angles. This saturation is evident in Supplementary Fig. S8b, in which we plot the

amplitude, $K(\alpha)$. We should again note that, in these numerical simulations, we neglected the bending effects, which are more pronounced at large tapering angles and large degrees of confinements. In order to capture the saturation of $K(\alpha)$ to a finite value for large angles and to reproduce $K(\alpha) \approx \tan\alpha$ for small angles, we choose $\gamma(\alpha) \approx (\tan\alpha)^{-11/14}$, which yields

$$K(\alpha) \approx [k + (\tan\alpha)^{-3/14}]^{-14/3}, \qquad \textbf{(71)}$$

where k is a numerical constant that can be obtained by the fit to experimental data.

In Supplementary Fig. S9a, we verify the above approximation by plotting the experimental data (*see* Fig. 5b) in the form $(\Delta P_{max}/E_0)^{-3/14}$ (D_0/d_c) as a function of $(\tan\alpha)^{-3/14}$. Fit of the function $[K(\alpha)]^{-3/14}$ by a linear dependence (predicted as in equation (71)) yields a best fitting parameter $k = 0.72 \pm 0.02$. In Supplementary Fig. S9b, we present the log-log plot of the experimental $\Delta P_{max}/E_0$ data rescaled with $K(\alpha)$ for $k = 0.72$, and observe that data for all angles collapse on a single line with a slope 14/3 as predicted by equation (69).

6. Flow resistance in the weak deformation regime

Consider a microgel weakly deformed in the tapered zone under a small pressure difference, $\Delta P \ll E_0$, where E_0 is the modulus of the microgel. To calculate the flow resistance of the weakly deformed spherical gel, we integrate the reciprocal resistance of "pipes" of diameter ξ_0, over the concentric rings (*see* Supplementary Fig. S10) of length $L(r) = 2\,[(D_0/2)^2 - r^2]^{1/2}$ and cross-sectional area $dA = 2\pi\, r\, dr$ (in the range $0 < r < D_0/2 - \delta$), *i.e.*,

$$\frac{1}{R} = \frac{\pi}{128}\frac{\xi_0^2}{\eta}\int\frac{\mathrm{d}A}{L(r)} = \frac{\pi}{128}\frac{\xi_0^2}{\eta}\int_0^{D_0/2-\delta}\frac{2\pi\, r\,\mathrm{d}r}{2\sqrt{(D_0/2)^2-r^2}}$$

$$= \frac{\pi^2}{256}\frac{\xi_0^2}{\eta}\left[D_0 - 2\sqrt{\delta(D_0-\delta)}\right] = \left(\frac{\pi}{16}\right)^2\frac{\xi_0^2}{\eta}\left[D_0 - L\left(\frac{D_0}{2}-\delta\right)\right], \qquad \textbf{(72)}$$

where $L(D_0/2-\delta)$ is the length of the contact zone $L(D_0/2-\delta) = 2\sqrt{\delta(D_0-\delta)}$. The resistance R

calculated in equation (72) is inversely proportional to the thickness of the caps $D_0 - L(D_0/2 - \delta)$. At small deformations ($\delta \ll D_0$) the flow resistance is equal to that of the unperturbed microgel

$$R \approx R_0 = (16/\pi)^2 \eta/(\xi_0^2 D_0) \qquad \text{for } \delta \ll D_0 \tag{73}$$

and is therefore constant independent of compression δ and pressure difference ΔP. This implies that most of the fluid flows through the whole microgel rather than through the small volume in the vicinity of the compressed microgel section of size $L(D_0/2 - \delta)$. The high resistance of longer pores (with length ~ $D_0 \gg L(D_0/2 - \delta)$) that pass through the central section of the microgel is compensated by the smaller number of such pipes. Thus we conclude that at small pressure difference, $\Delta P \ll E_0$, the flux Q of water through the microgel is linearly proportional to the pressure difference

$$Q \approx \Delta P/R_0\,, \tag{74}$$

in good agreement with numerical calculations (red line with unit slope in Supplementary Fig. S7d) and experiments (red line with unit slope in Fig. 3b).

Balance of elastic and osmotic stresses at undeformed equilibrium state leads to[9] $E_0 \approx 2kT/\xi_0^3$. For the Young's modulus E_0 = 2570 Pa of the microgel used in the flow experiments, and $kT \approx 4.11\cdot10^{-3}$ Pa·μm^3 at room temperature, we estimate the pore size in undeformed microgel to be $\xi_0 \approx 15$ nm. Substituting this value into the equation (73) for the resistance R_0 of the undeformed gel of diameter $D_0 \approx$ 100 μm with viscosity $\eta \approx 0.9\cdot10^{-3}$ Pa·s for water at room temperature, we obtain $R_0 \approx 1$ Pa·s/ μm^3. This estimate is in excellent agreement with the experimental results – the best linear fit to the data (red line in Fig. 3b) gives

$$R_0 = 1 \text{ Pa·s/μm}^3. \tag{75}$$

Supplementary References

1. Li, Y., Kumacheva, E., Ramachandran, A. & Li Y Ramachandra A, K. E. The motion of a microgel in an axisymmetric constriction with a tapered entrance. *Soft Matter* **9,** 10391–10403 (2013).

2. Chapra, S. C. & Canale, R. P. *Numerical Methods for Engineers*. (McGraw-Hill Science/Engineering/Math, 2009).

3. Aris, R. On the dispersion of a solute in a fluid flowing through a tube. *Proc. R. Soc. Lond. A* **235,** 67–77 (1956).

4. Landau, L. D., Pitaevskii, L. P., Kosevich, A. M. & Lifshitz, E. M. in *Course Theor. Phys.* Volume 7 (Elsevier, 1986).

5. Bruus, H. *Theoretical microfluidics*. (Oxford University Press, 2007).

6. Tan, S. H., Nguyen, N.-T., Chua, Y. C. & Kang, T. G. Oxygen plasma treatment for reducing hydrophobicity of a sealed polydimethylsiloxane microchannel. *Biomicrofluidics* **4,** 032204 (2010).

7. Flory, P. J. & Rehner, J. Statistical mechanics of cross-linked polymer networks II. swelling. *J. Chem. Phys.* **11,** 521–526 (1943).

8. Gennes, P.-G. De. *Scaling concepts in polymer physics*. (Cornell University Press, 1979).

9. Rubinstein, M. & Colby, R. H. *Polymer Physics*. (Oxford University Press, 2003).

10. Pekarski, P., Tkachenko, A. & Rabin, Y. Deformation-induced anomalous swelling of topologically disordered gels. *Macromolecules* **27,** 7192–7196 (1994).
